# Neural circuit mechanisms underlying context-specific halting in *Drosophila*

**DOI:** 10.1038/s41586-024-07854-7

**Published:** 2024-10-02

**Authors:** Neha Sapkal, Nino Mancini, Divya Sthanu Kumar, Nico Spiller, Kazuma Murakami, Gianna Vitelli, Benjamin Bargeron, Kate Maier, Katharina Eichler, Gregory S. X. E. Jefferis, Philip K. Shiu, Gabriella R. Sterne, Salil S. Bidaye

**Affiliations:** 1 Max Planck Florida Institute for Neuroscience, Jupiter, FL USA; 2 International Max Planck Research School for Synapses and Circuits, Jupiter, FL, USA; 3 Florida Atlantic University, Boca Raton, FL, USA; 4 Drosophila Connectomics Group, Department of Zoology, University of Cambridge, Cambridge, UK; 5 Neurobiology Division, MRC Laboratory of Molecular Biology, Cambridge, UK; 6 Department of Molecular and Cell Biology and Helen Wills Neuroscience Institute, University of California, Berkeley, CA USA; 7 Present Address: Department of Biomedical Genetics, University of Rochester Medical Center, Rochester, NY USA

**Keywords:** Neural circuits, Reflexes, Spinal cord, Network models

## Abstract

Walking is a complex motor programme involving coordinated and distributed activity across the brain and the spinal cord. Halting appropriately at the correct time is a critical component of walking control. Despite progress in identifying neurons driving halting^1–6^, the underlying neural circuit mechanisms responsible for overruling the competing walking state remain unclear. Here, using connectome-informed models^7–9^ and functional studies, we explain two fundamental mechanisms by which *Drosophila* implement context-appropriate halting. The first mechanism (‘walk-OFF’) relies on GABAergic neurons that inhibit specific descending walking commands in the brain, whereas the second mechanism (‘brake’) relies on excitatory cholinergic neurons in the nerve cord that lead to an active arrest of stepping movements. We show that two neurons that deploy the walk-OFF mechanism inhibit distinct populations of walking-promotion neurons, leading to differential halting of forward walking or turning. The brake neurons, by constrast, override all walking commands by simultaneously inhibiting descending walking-promotion neurons and increasing the resistance at the leg joints. We characterized two behavioural contexts in which the distinct halting mechanisms were used by the animal in a mutually exclusive manner: the walk-OFF mechanism was engaged for halting during feeding and the brake mechanism was engaged for halting and stability during grooming.

## Abstract (web summary)

Two halting mechanisms, ‘walk-OFF’ and ‘brake’, are shown to be engaged by distinct neural circuits in *Drosophila*, in a context dependent manner.

## Main

Walking recruits distributed neural activity across the brain^6,10–12^ and spinal cord^13,14^ or nerve cord^15,16^. When an animal halts, the net output of this distributed neural activity notably changes to arrest leg stepping movements. Moreover, depending on the behavioural context, animals halt in different context-appropriate ways. Whereas a few specific neuronal types driving halting have been recently described^1,2,4–6,17,18^, how walking-related neural activity changes to allow halting in a manner that is both mechanically stable and appropriate to the behavioural context is at present unclear.

## Identification of halt neurons

**Fig. 1:**
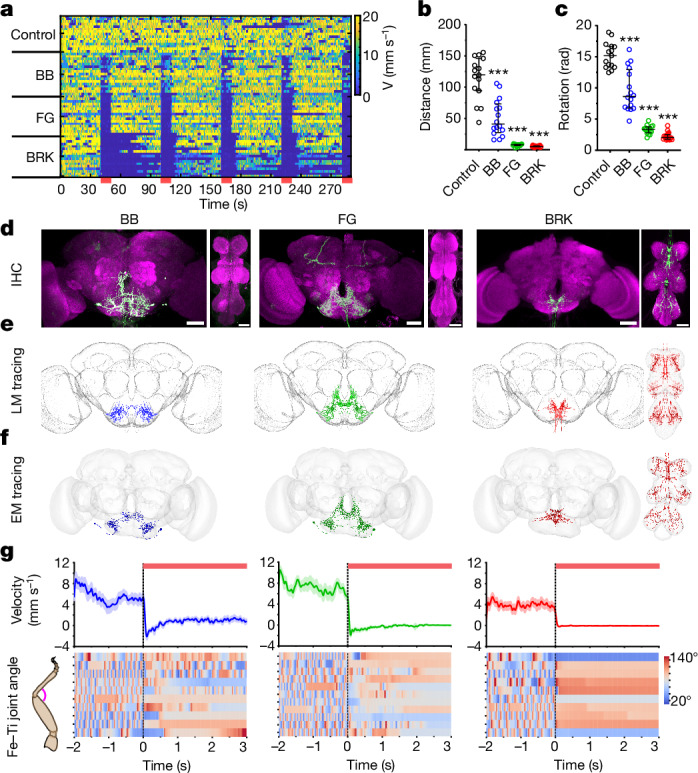
Identification of halt neurons. **a**–**c**, Translational velocity heatmap (**a**) (every row shows velocity of single fly, red bars indicate optogenetic stimulation), trial averaged travelled distance (**b**, median ± interquartile) and rotation (**c**, median ± interquartile) of free-walking control flies and flies expressing CsChrimson in halting neurons (BB, FG and BRK). *n* = 15–18 flies per genotype, Mann–Whitney test compared to genetic-background control (***P < 0.001). **d**–**f**, Immunohistochemistry (IHC) images of the selected genetic drivers (CsChrimson-mVenus, green; neuropil, magenta; scale bars, 50 µm) (**d**), light microscopy (LM)-based segmentation (**e**) and electron microscopy (EM)-based segmentation (**f**) of the BB (left), FG (middle) and BRK (right) halt neurons. **g**, Forward velocity (top, mean ± s.e.m) and Fe–Ti joint angle heatmap (bottom, every row represents a different fly) of tethered walking flies during optogenetic stimulation of BB (left), FG (middle) and BRK (right) neurons. Here, 3 s of stimulation (top red bar) starts at dotted vertical line in velocity plots (also Supplementary Video 1). Supplementary Table 1 shows full experimental genotypes and exact sample sizes. Source Data

**Extended Data Fig. 1:**
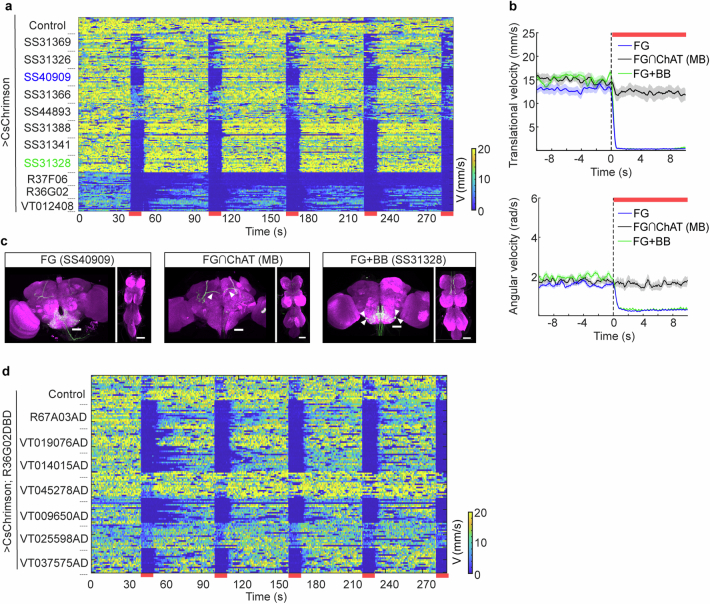
Neural activation screen to identify halting neurons. **a**. Translation velocity heat map of free-walking, control flies and broad lines expressing CsChrimson. Red bars at bottom of heatmap indicate 5 trials of 10 s red light driven optogenetic stimulation. n = 16 flies per genotype. **b**. From (a), showing trial averaged (mean ± s.e.m.) translational velocity (top) and angular velocity (bottom) of FG (SS40909), FG∩ChAT (MB), FG + BB (SS31328). **c**. Immunohistochemistry images of genetic reagents used in (**b**); white arrowheads show mushroom bodies (MB) (CsChrimson-mVenus: green, neuropil: magenta, scale bars: 100 μm (brain), 50 μm (VNC)). **d**. Translation velocity heat map (every row shows velocity of single fly) of free-walking flies with a focus on sparse genetic lines labelling BRK. Red bars at bottom of heatmap indicate five trials of 10 s red light driven optogenetic stimulation. Source Data

**Extended Data Fig. 2:**
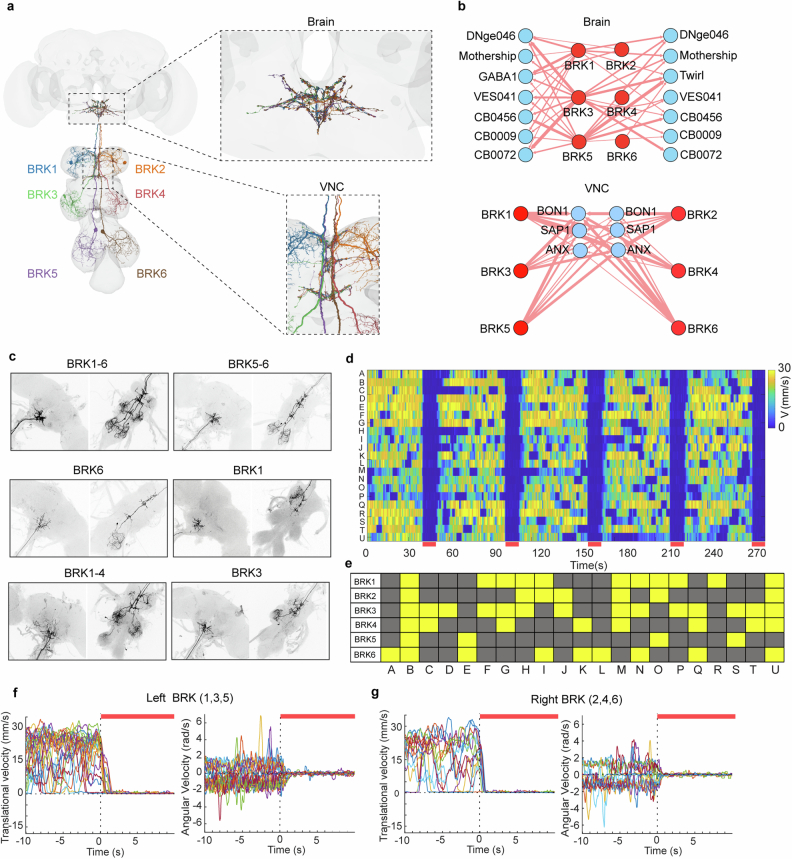
Activating any BRK neuron is sufficient to drive halting. **a**. EM segmentation of all 6 BRK neurons. Insets show BRK axonal projections in the brain (top) and VNC (bottom). **b**. Connectome-based wiring diagram showing BRK major post-synaptic partners in the brain and VNC. Arrow thickness indicates synaptic strength (top: 5-100 synapses; bottom: 5-400 synapses); red indicates predicted excitatory connections. **c**. Stochastic expression of CsChrimson-mVenus in distinct subsets of BRK neurons. **d**. Translation velocity heat map of free-walking flies (every row shows velocity of single fly labelled from A-U) with different BRK segments stochastically labelled. Red bars at bottom of heat map indicate 5 trials of 10 s red light driven optogenetic stimulation. **e**. Each column represents stochastic CsChrimson expression (for same flies in (**d**)) in the indicated BRK neurons in a single fly (yellow: show expression; grey: absent/no expression). Translational angular (left) and angular velocity (right) of individual, free-walking flies with only left-BRK (1,3,5) (**f**) and only right-BRK (2,4,6) (**g**) optogenetically activated (red bars) using CsChrimson. Data in (**f**) and (**g**) are part of the dataset shown in (**d**). Supplementary Table 1 shows full genotypes and exact sample sizes. Source Data

To identify neurons involved in halting in *Drosophila*, we optogenetically activated specific neurons that innervate the suboesophageal zone (SEZ) of the fly brain^19,20^, a region that houses the neurons close to and including the descending output of the brain^21^ (analogous to mammalian brainstem^22^). Through this neural-activation screen (Methods), we identified 11 genetic drivers that were both relatively sparse and caused halting without gross motor deficits (Extended Data Fig. 1a). Among these lines, we could unambiguously pinpoint three causal neurons for halting, which we refer to as Foxglove (FG), Bluebell (BB) and Brake (BRK) (Fig. 1). We identified split-Gal4 reagents that targeted FG, BB and BRK with little to no ectopic expression and drove robust halting as reflected by decrease in walking velocity, distance and rotation (Fig. 1a–d and Supplementary Tables 1 and 2 for all genotypes). Using connectome parsing tools^23–26^ (Methods), we identified these neurons in the brain^8,9^ and nerve-cord^27–29^ connectomes (Fig. 1e,f and Supplementary Table 3). Both FG and BB (anatomy described in ref. ^20^) are SEZ neurons that stochastically descend until the anterior tip of the nerve cord. The FG line has weak ectopic expression in the mushroom body neuropil that does not contribute to the halting phenotype (Extended Data Fig. 1b,c). The BB line also labels (often unilaterally) an additional neuron previously described as ‘bluebird’^20^ (Fig. 1d). BRK is a new neuronal type discovered in this work and drives the strongest halting phenotype (Fig. 1a–c and Extended Data Fig. 1d). BRK comprises six ascending neurons with their somata in each of the six leg-segment-specific neuromeres of the ventral nerve cord (VNC) and axonal projections to the brain SEZ region (Fig. 1d–f and Extended Data Fig. 2a,b). By activating subsets of BRK neurons, we found that activating any of the BRK neurons is sufficient to drive halting (Extended Data Fig. 2c–g). Consistent with this, in the connectome, we found that although each BRK gets distinct inputs, all the six BRK share common major outputs in their two output zones, the tectulum^30^ of the VNC and the SEZ of the brain (Extended Data Fig. 2a,b).

**Extended Data Fig. 3:**
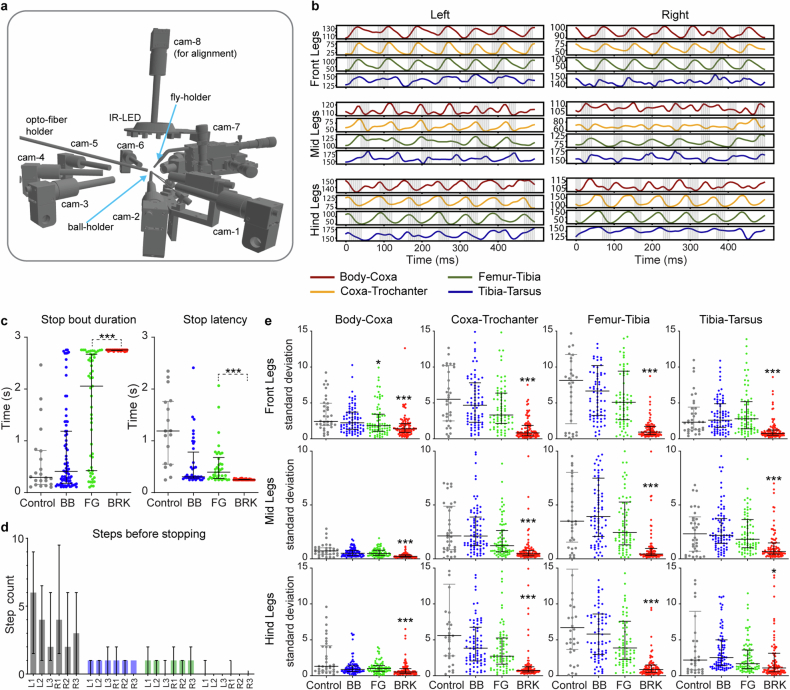
High resolution 3D leg kinematics analysis. **a**. Schematic of the eight-camera setup. A fly tethered to a pin at its thorax is mounted on an air supported ball at the center. The fly is illuminated using a custom-made IR-LED ring from above. Seven synchronized high-speed cameras image the fly from different sides. The top-down camera is used to guide the alignment of the fly on the surface of the ball such that it is centered. Two motion sensors track the rotations of the ball allowing the reconstruction of the fly’s virtual trajectory and velocity. Red light for optogenetic activation is supplied through an optical fibre in front of the fly. **b**. Example traces of the four flexion angles (body-coxa, coxa-trochanter, femur-tibia and tibia-tarsus) for all 6 legs in a tethered walking fly. Grey shading indicates swing phase of the step cycle. **c**. Stopping bout durations (left) (n = 22–75 bouts per genotype, median ± interquartile) and stopping latency (right) (n = 17–40 trials per genotype, median ± interquartile) during optogenetic activation of BB, FG and BRK. Mann-Whitney Test to compare between FG and BRK (***p < 0.001). **d**. Number of steps before the flies stop upon optogenetic activation of BB, FG and BRK, compared to control flies **e**. Standard deviation of all flexion angles calculated over the whole light-ON period, for the respective genotypes. Kruskal–Wallis test followed by Dunn’s multiple comparison with control (median ± interquartile, *p < 0.05, ***p < 0.001). Supplementary Table 1 shows full genotypes and exact sample sizes. Source Data

Analysis of walking velocities on FG, BB or BRK stimulation (Fig. 1a–c) hinted at potential differences in the halting phenotypes. To precisely quantify such differences, we performed, high-resolution three-dimensional (3D) leg kinematics analysis (Methods, Extended Data Fig. 3a,b and Supplementary Video 1). BRK drove low-latency, long-duration halting bouts (Extended Data Fig. 3c) with joint angles locked in a specific position for the entire halt duration (Fig. 1g and Extended Data Fig. 3e). By contrast, FG and BB induced halts were interspersed with leg movements. FG drove long halting bouts like BRK but still allowed brief repositioning of legs (Fig. 1g and Extended Data Fig. 3e). BB drove stationary periods interspersed with slow walking and allowed more leg movements (Fig. 1g and Supplementary Video 1). Unlike BRK, both FG and BB stimulated flies often took a few steps before halting (Extended Data Fig. 3d).

Defining the genetic drivers and connectome identities of ‘halt’ neurons driving distinct types of halting, allowed us to next address their interaction with the walking-promoting circuits.

## Diversity in walk–halt interactions

**Extended Data Fig. 4:**
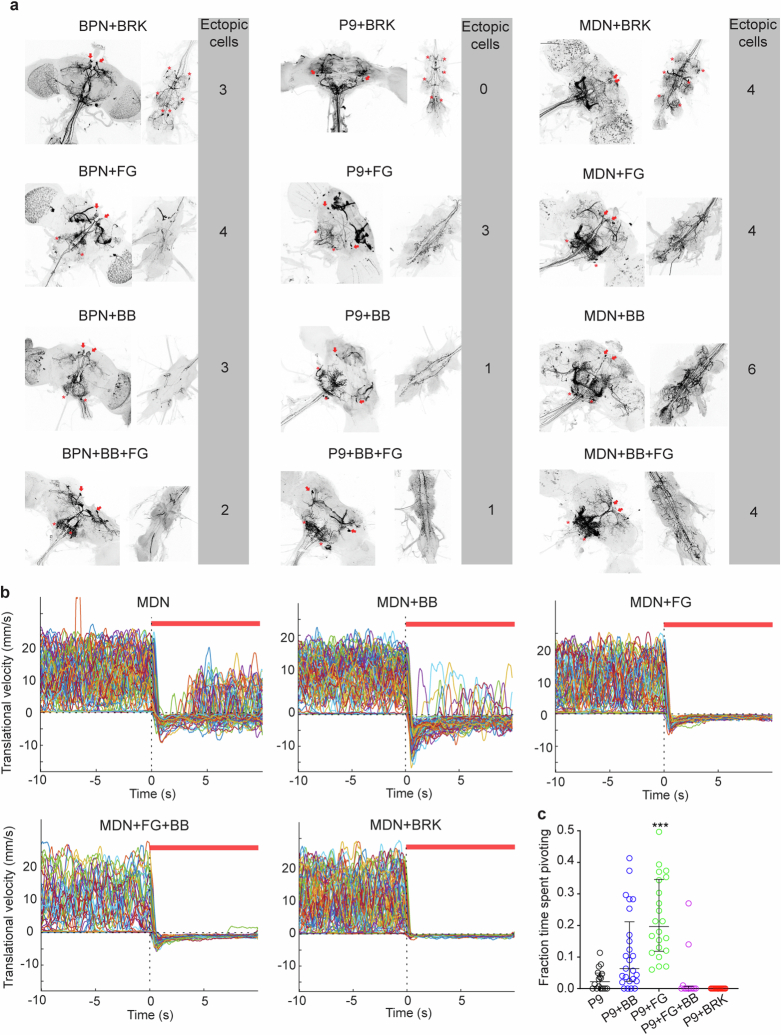
Walk-Halt Co-activation. **a**. Light microscopy images of combined split-Gal4 lines comprising walk neurons (P9, BPN, MDN) and halt neurons (BRK, FG, BB, and the FG + BB line). Arrows indicate walk neurons, and asterisks show halt neurons. Ectopic expression is indicated as cell numbers. **b**. Per-trial translational velocity across all flies (n = 10-16 flies per genotype, 4 trials per fly) with MDN coactivated with BB, FG, BB + FG, or BRK. 10 s light stimulation (red bars) starts at vertical stippled line in velocity plots. **c**. Fraction of time free-walking flies spent pivoting of upon P9 co-activation with BB, FG, FG + BB, or BRK. Dots indicate individual flies. n = 12-25 flies per genotype, median ± interquartile range. Kruskal-Wallis test followed by Dunn’s multiple comparison with genetic-background control (***p < 0.001). See Supplementary Video 2 and 3. Supplementary Table 1 shows full genotypes and exact sample sizes. Source Data

To address if and how the newly identified halt neurons interact with walking-promoting pathways, we leveraged genetic tools targeting walking-promoting (‘walk’) neurons characterized in our previous work, namely P9 (DNp09) that drives forward turning^19,21^, BPN (bolt protocerebral neuron) that drives straight forward walking^19^ and MDN (moonwalker descending neuron) that drives backward walking^31^. We combined split-Gal4 reagents for the walk and halt neurons and confirmed opsin expression in the desired neurons (Extended Data Fig. 4a). Here, in addition to the three halt neuron driver lines, and to confirm the role of BB, we used another driver line (SS31328, Extended Data Fig. 1b,c) that targets FG + BB without any ectopic expression. Using these tools, we performed all possible combinations of ‘walk + halt’ coactivations.

**Fig. 2:**
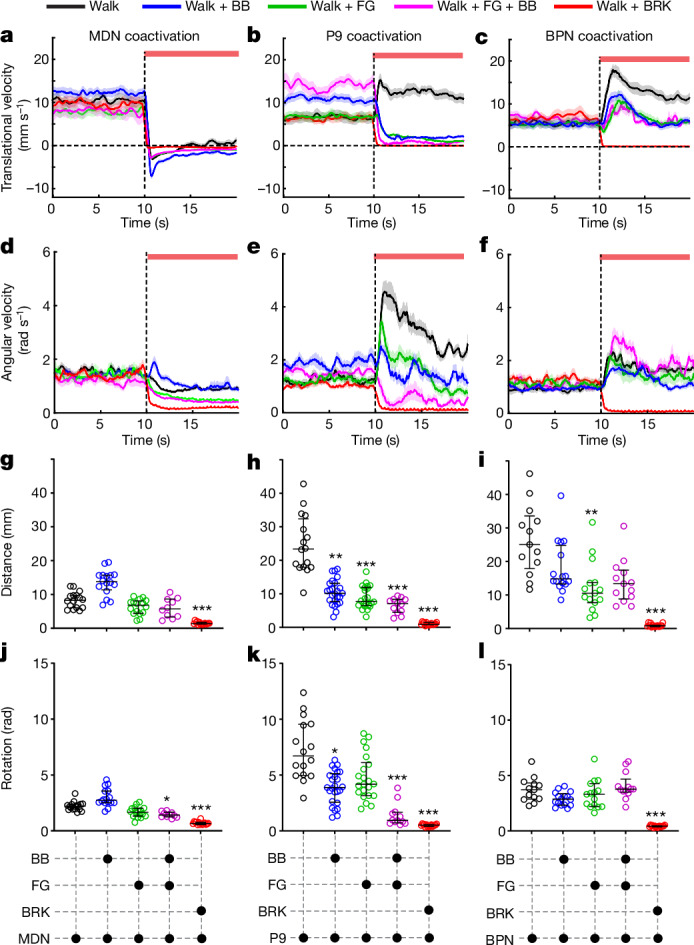
Walk–halt interactions. **a**–**l**, Trial averaged (mean ± s.e.m) translational velocity (**a**–**c**), angular velocity (**d**–**f**), distance covered in 2 s after stimulation onset (**g**–**i**), median ± interquartile) and rotations in 2 s after stimulation onset (**j**–**l**, median ± interquartile) of free-walking flies with pairs of walk and halt neurons optogenetically stimulated. Stimulation (red bar) starts at dotted vertical line in velocity plots. Each graph represents quantification for MDN (**a**,**d**,**g**,**j**), P9 (**b**,**e**,**h**,**k**) and BPN (**c**,**f**,**i**,**l**) coactivation experiments. *n* = 10–25 flies per genotype, Kruskal–Wallis test followed by Dunn’s multiple comparison with genetic-background control (***P < 0.001, **P < 0.01, *P < 0.05) (also Supplementary Videos 2 and 3). Supplementary Table 1 shows full experimental genotypes and exact sample sizes. Source Data

BRK stimulation completely suppressed all walking commands (forward, backward and turning), suggesting that it drives a dominant halting mechanism (Fig. 2). By contrast, FG and BB activation affected the walk neuron phenotypes with varying degrees and specificity, suggesting distinct halting mechanisms being deployed in these cases.

During coactivation with MDN, unlike BRK, FG and BB did not suppress backward walking (Fig. 2a,d,g,j). However, the intermittent switches to forward walking observed during continuous MDN stimulation (Methods) were absent (FG + MDN) or markedly reduced (BB + MDN) as seen in the individual trial velocity traces (Extended Data Fig. 4b and Supplementary Video 2). This suggests that FG and BB predominantly suppress forward walking.

Consistent with this, when FG or BB were coactivated with P9, the flies showed a marked reduction in forward velocity (Fig. 2b,h). BB + P9 or FG + BB + P9 coactivated flies also showed a corresponding decrease in angular velocity compared to P9-stimulated flies (Fig. 2e,k). However, FG + P9 coactivated flies still showed a large increase in angular velocity at stimulation onset. We found that while the BB + P9 or FG + BB + P9 flies were predominantly halting during the optogenetic stimulation, FG + P9 flies were pivoting on the spot (Extended Data Fig. 4c and Supplementary Video 3). These observations revealed an unexpected modularity in the P9 downstream pathway into two components: a forward walking component that is suppressed by both FG and BB and a turning component that is suppressed by BB. As the FG + BB line (targets FG and BB) has a similar phenotype as the BB line (targets BB and ‘bluebird’), we conclude that BB and not bluebird is responsible for the forward turning suppression.

When FG or BB was coactivated with BPN, the flies still increased forward walking during optogenetic stimulation (Fig. 2c). Whereas this increase in forward walking tended to be smaller than that of activation of BPN alone, only in the case of FG could we see a statistically significant difference in the distance quantification (Fig. 2i). Although BPN + FG + BB coactivated flies decreased forward walking it was interspersed with intermittent jumps possibly due to ectopic expression in the VNC (Extended Data Fig. 4a) rendering the distance quantification unreliable (Fig. 2i). On the basis of these results, we conclude that FG can partially suppress the BPN driven forward walking.

To summarize, we found that (1) BRK suppresses all walking commands, (2) FG specifically suppresses forward walking components of P9 and BPN (partially) and (3) BB predominantly suppresses forward turning induced by P9. The diversity in these walk–halt interactions suggests that the neural targets and mechanisms deployed by the three halting pathways (FG, BB, BRK) are at least partially distinct.

## The walk-OFF mechanism

**Extended Data Fig. 5:**
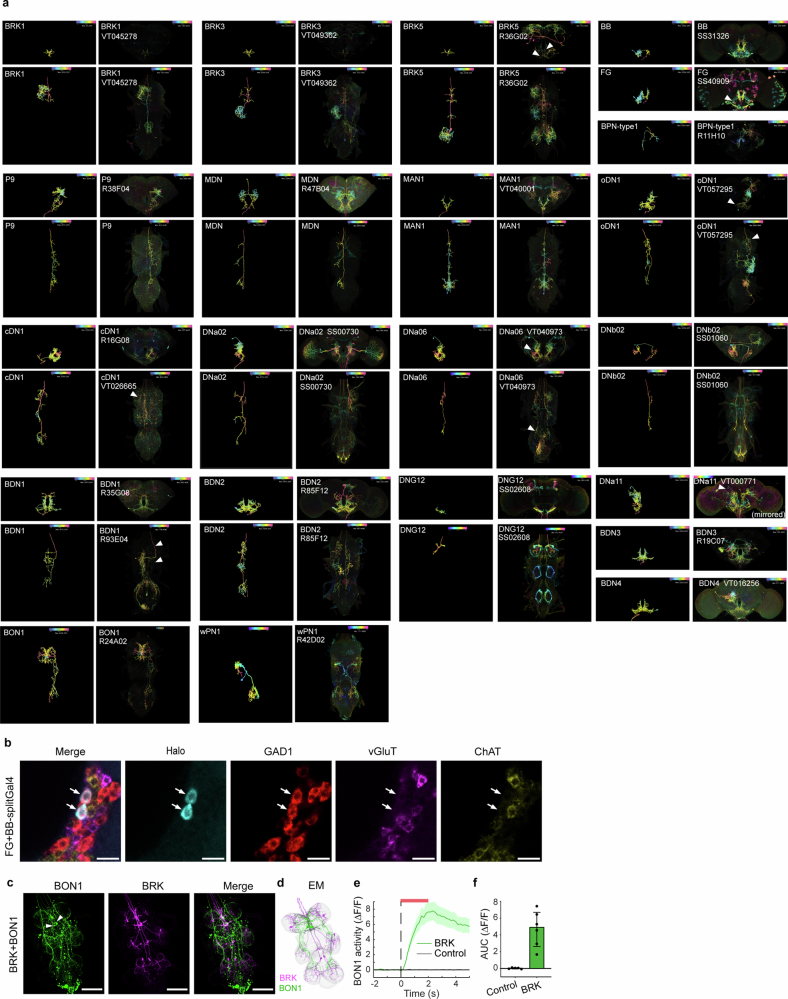
Validating morphology and neurotransmitter profiles across light and electron microscopy data. **a**. MCFO-colormip (color-coded maximum intensity projection) comparison (brain and VNC) for neurons of major interest in this study, grouped by halt and walk neuronal types, and by their major downstream synaptic partners. Nomenclatures to annotations given in light- and electron-microscopy datasets. White arrowheads show neuron of interest. **b**. Neurotransmitter profile for the combined FG + BB split-Gal4 using FISH. Arrows indicate BB/FG soma. **c**. Light-microscopy images of genetic line co-labelling BRK and BON1; (GCaMP6s: green, ChrimsonR-mCherry: magenta, scale bars: 100 μm, white arrowheads indicate BON1 soma). **d**. EM segmentation of BRK and BON1 in the VNC. **e**. BON1(Brake Output Neuron 1) activity (GCaMP6s) in explant brains without or with BRK activated (ChrimsonR). 2 s optogenetic activation (red bar) starts at vertical stipple line. n = 5-6 brains per genotype, mean ± s.e.m. **f**. Area under the curve (AUC) for 0-2 s after stimulation onset both in control and BRK activated groups from (**e**), median±interquartile, dots indicate number of brains. Source Data

**Extended Data Fig. 6:**
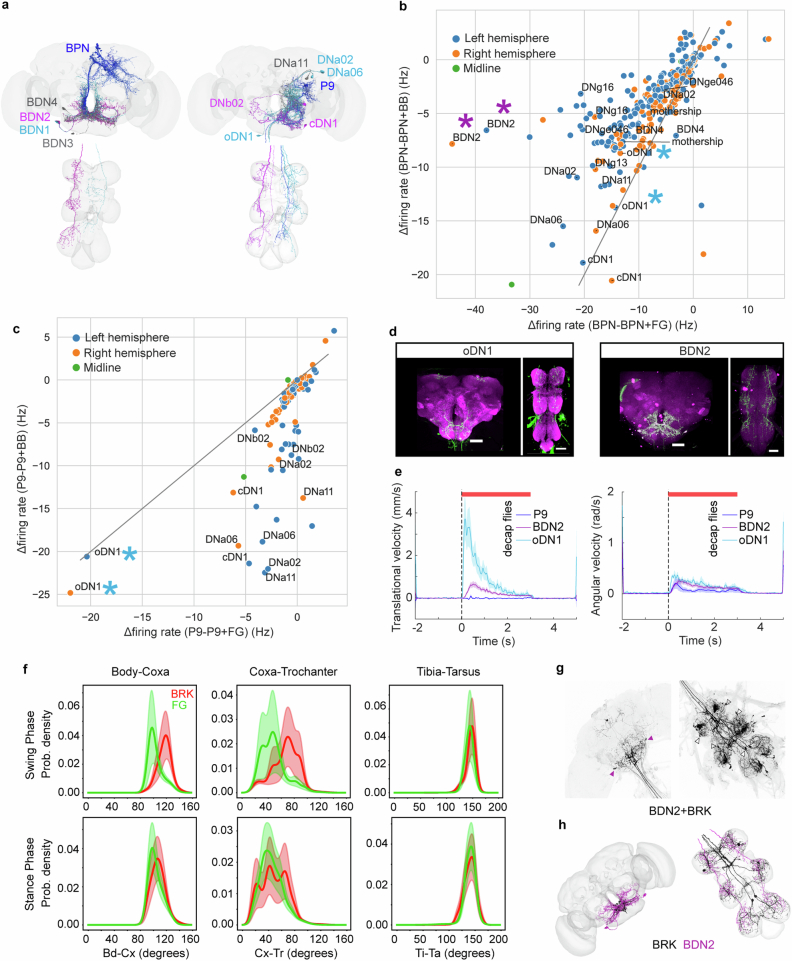
Analysis pertaining to Walk-OFF and Brake mechanisms. **a**. DNs recruited by BPN and P9 (magenta: contralateral descending; blue: ipsilateral descending; grey: unidentified descending arbors). **b,c**. Scatter plot showing DNs downstream of (**b**) BPN and (**c**) P9 differentially affected by FG versus BB. **d**. Expression pattern of split-Gal4 lines covering oDN1 and BDN2. (CsChrimson-mVenus: green, neuropil: magenta, scale bars: 50 μm). **e**. Trial averaged translational velocity (left) and angular velocity (right) of decapitated, tethered flies while activating P9, oDN1, or BDN2. 3 s light stimulation (red bars) starts at vertical stippled lines in velocity plots. n = 7-10 flies per genotype, mean ± s.e.m. **f**. Probability density of front leg joint angle (body-coxa, coxa-trochanter and tibia-tarsus) if BRK or FG was optogenetically activated during swing phase (top) or during stance phase (bottom). n = 7-33 trials averaged across 6-17 flies on the ball. **g**. Light microscopy images of combined split-Gal4 lines for co-labelling BRK and BDN2. Magenta arrowheads in the brain indicate BDN’s soma (left), empty arrowhead in the VNC indicate BRK’s soma (right). **h**. EM segmentation of BRK and BDN2 in the brain (left) and VNC (right). Supplementary Table 3 describes all connectome IDs. Supplementary Table 1 shows full genotypes and exact sample sizes. Source Data

Guided by the whole fly brain connectome and the FlyWire toolkit^26^, we asked if and where the halting and walking pathways might converge. We identified, annotated and proofread all the walk and halt neuronal types and their downstream partners in the connectome^8,9^ (Extended Data Fig. 5a and Supplementary Table 3). We found no direct connections between the walk and halt neurons. However, all the walk and halt neurons share a common output zone, the SEZ, where they could converge on same output neurons. Even though MDN and P9 are descending neurons (DNs) that project from the brain to the VNC, they have collaterals in the SEZ through which they recruit other neurons including DNs. Decapitated MDN flies, in which these SEZ collaterals are eliminated, show backward walking on optogenetic stimulation^32^ indicating that the VNC circuit recruited by MDNs is sufficient for driving the phenotype. However, decapitated P9-activated flies do not show the phenotypic forward turning (Extended Data Fig. 6e). This implies that the other DNs recruited by P9 SEZ collaterals (Extended Data Fig. 6a) are critical for the phenotype. Indeed, some P9 downstream DNs (DNa02 and DNb02) were previously implicated in turning^15,33,34^. BPN is a higher brain neuron^19^ that directly recruits other DNs through SEZ outputs. We therefore focused on identifying whether halting pathway projections to the SEZ impinge on DNs recruited by P9 and BPN.

To comprehensively analyse interaction between halting and walking pathways, we used a connectome-constrained whole-brain spiking-network model to reproduce the behavioural coactivation experiments in silico. The model (ref. ^7^ and Methods), uses the entire connectivity matrix from the FlyWire data^8^, and assigns positive or negative weight to the connection based on synapse number and predicted neurotransmitter^35^. We validated the neurotransmitter predictions for the halt neurons and confirmed that FG and BB are γ-aminobutyric acid (GABA)ergic (inhibitory) neurons, whereas BRK is an excitatory (potential cholinergic) neuronal type (Extended Data Fig. 5b–f).

**Fig. 3:**
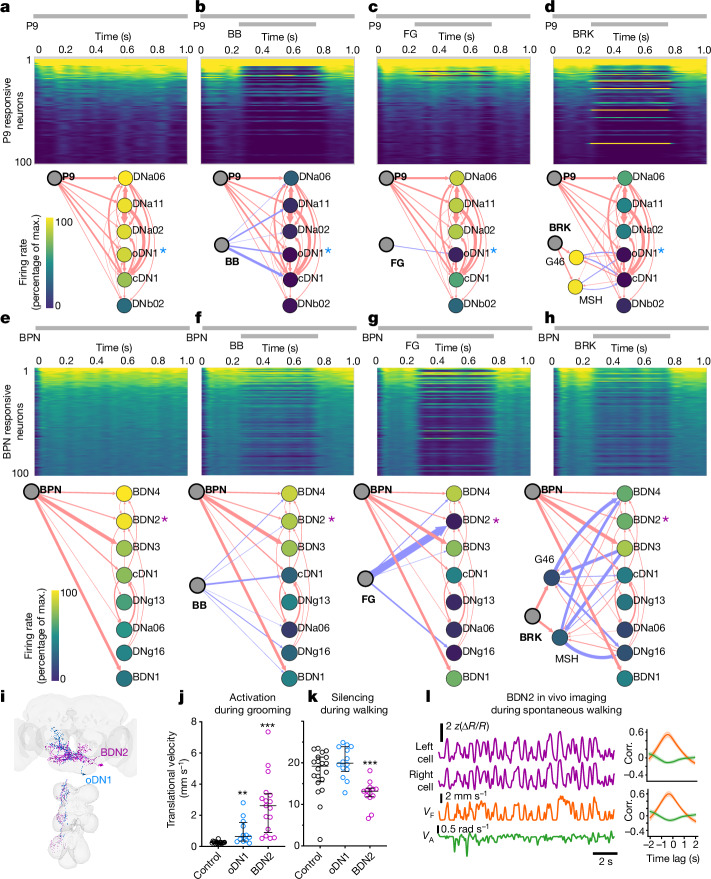
Modelling and functional data uncover critical walking-promotion nodes inhibited by halting pathways in the walk-OFF mechanism. **a**–**h**, Simulation results for activating P9 (**a**), P9 + BB (**b**), P9 + FG (**c**), P9 + BRK (**d**), BPN (**e**), BPN + BB (**f**), BPN + FG (**g**) and BPN + BRK (**h**), activation paradigm indicated with grey bars on top. Trial averaged firing rate heatmap of top 100 neurons responding to in silico P9 (**a**–**d**) or BPN (**e**–**h**) coactivation with halt neurons, is shown on top and connectome-based wiring diagram for strongly recruited DNs is shown on bottom. Nodes in the wiring diagrams are colour-coded based on firing rate in the full simulation as in the heatmaps; the colour scale is normalized to the maximum (max.) trial averaged firing rate achieved in the case of the respective walk neuron activation simulation. Red arrows indicate predicted excitatory connections, and blue arrows indicate predicted inhibitory connections; arrow width corresponds to synaptic count, and scales from five (thinnest) to 400 (thickest) synapses. Asterisks indicate neurons selected for further analysis in **i**–**l**. **i**, EM segmentation of oDN1 and BDN2 neurons. **j**, Trial averaged translational velocity of stationary grooming flies induced to walk on optogenetic activation of oDN1 or BDN2. *n* = 12–17 flies per genotype, median ± interquartile, Mann–Whitney test (**P < 0.01, ***P < 0.001). **k**, Trial averaged translational velocity of free-walking flies with oDN1 or BDN2 optogenetic silenced (GtACR1). *n* = 13–20 flies per genotype, median ± interquartile, Mann–Whitney test (***P < 0.001). **l**, BDN2 activity (top two traces corresponding to left and right BDN2, respectively) in a tethered fly spontaneously walking with forward velocity (*V*_F_) and angular velocity (*V*_A_), bottom traces. Right panels show cross-correlation (Corr.) of pooled data across three flies for activity of BDN2 left cell (top) and BDN2 right cell (bottom) with *V*_F_ (orange) and *V*_A_ (green) (Supplementary Video 4). Supplementary Table 1 shows full experimental genotypes and exact sample sizes. Source Data

Comparing responses across top 100 responding neurons in each in silico coactivation experiment (heatmaps in Fig. 3a–h, top) show that all three halt neurons inhibit different, partially overlapping subsets of neurons recruited by the walk pathways. Although the P9 pathway is more broadly and strongly inhibited by BB, the BPN pathway is more broadly and strongly inhibited by FG (Fig. 3a–h, top, and Extended Data Fig. 6b,c). This aligns with the corresponding behavioural phenotypes (Fig. 2).

We next focused on DNs strongly recruited by the walk pathways and generated synapse-weighted graphs to simultaneously visualize the connectivity and predicted firing rate (Methods and Fig. 3a–h, bottom). In the case of P9 coactivation with halt neurons, BB suppressed the firing rate of most of the DNs whereas FG specifically inhibited a single DN named oDN1 (Fig. 3b,c, bottom). The BB result is consistent with the complete suppression of P9 phenotype in behavioural experiments (Fig. 2b,e,h,k). Given that FG only suppressed the forward walking component of P9 phenotype (Fig. 2b,e,h,k), we infer that oDN1 is mainly relevant for driving forward velocity but not rotational velocity.

In the case of DNs recruited by BPN, neither FG nor BB inhibits the whole population (Fig. 3e–g, bottom). This could explain why we never saw a complete suppression of walking in corresponding behavioural experiments (Fig. 2c,f,i). To probe why FG has a stronger effect in behavioural experiments, we compared the neurons that are differentially affected by FG versus BB. BDN2 stood apart as a DN specifically and strongly inhibited by FG (Fig. 3g, bottom, and Extended Data Fig. 6b).

Taken together, the modelling and behavioural data show that FG and BB are positioned to directly inhibit critical nodes in the P9 and BPN walking-promotion pathways and decrease forward and/or rotational walking velocity. We term this the walk-OFF mechanism. Furthermore, oDN1 and BDN2 (Fig. 3i) stood apart as important nodes to mediate differences in BB and FG effects on the walking pathways (Fig. 3a–h and Extended Data Fig. 6b,c). We therefore generated split-Gal4 drivers (Extended Data Fig. 6d) to specifically target these neurons. Optogenetically activating oDN1 and BDN2 drove walking initiation, with BDN2 showing a much stronger phenotype (Fig. 3j). Even decapitated oDN1 and BDN2 activated flies initiated robust forward walking (Extended Data Fig. 6e) suggesting that unlike P9, the VNC outputs of these DNs are sufficient for forward walking. However, on optogenetic silencing, only BDN2-silenced flies showed a reduction in forward velocity (Fig. 3k). This suggests that BDN2 might be critical during spontaneous walking, whereas oDN1 could be redundant or recruited only in specific contexts. Indeed, on in vivo imaging, we found that BDN2 activity is strongly correlated to forward (but not angular) velocity of the fly (Fig. 3l and Supplementary Video 4). Given that BDN2 is strongly inhibited by FG and not by BB (Fig. 3f,g), this could explain why FG drives stronger halting in spontaneously walking flies (Fig. 1a–c).

In silico coactivating BRK ascending projections, along with P9 (Fig. 3d) and BPN (Fig. 3h) showed that while BRK is excitatory, it recruits other predicted inhibitory neurons in the SEZ to suppress walking-promotion DNs. When coactivated with P9, BRK inhibits many of the critical walking-promotion DNs, including oDN1. However, when coactivated with BPN, BRK inhibits only a subset of DNs, with BDN2 being weakly inhibited. This is in contrast to the strong walking suppression observed in the corresponding behavioural experiments (Fig. 2). Crucially, BRK activation suppressed backward walking driven by MDN, whose descending output in the VNC is sufficient to drive backward walking (Fig. 2a,d,g,j). This suggests that, in addition to inhibiting some walking-promoting pathways in the brain (walk-OFF mechanism), BRK probably acts in the VNC to drive halting.

## The ‘brake’ mechanism

**Fig. 4:**
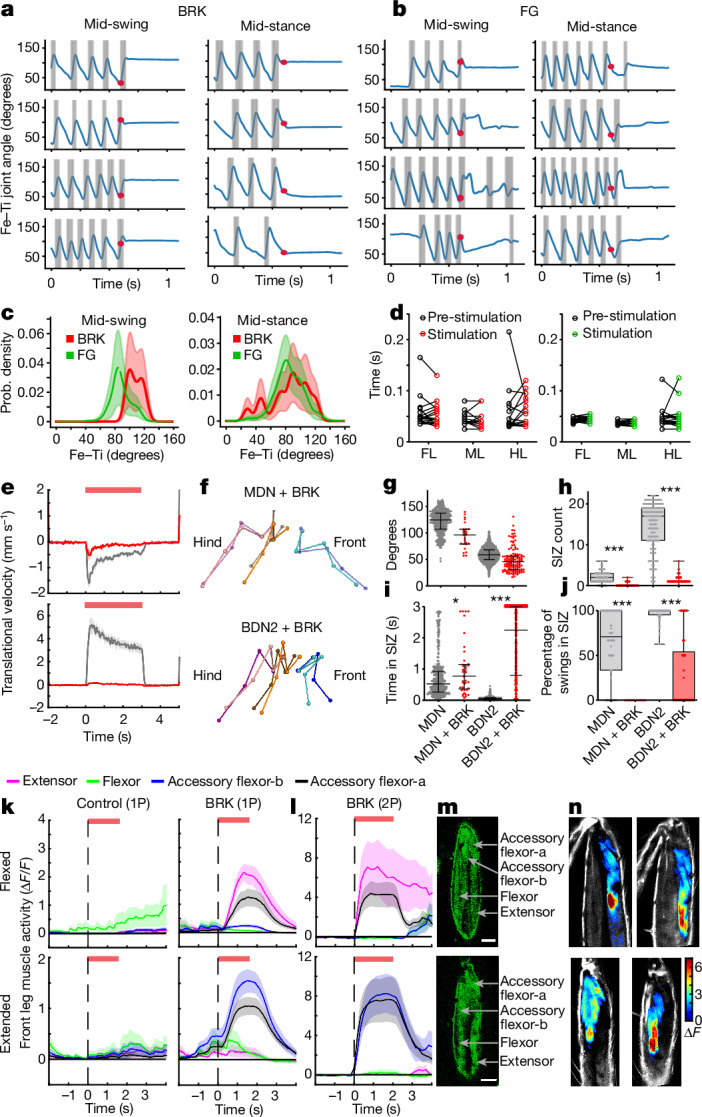
VNC-specific halting pathway for increasing resistance to leg movements in the brake mechanism. **a**,**b**, Front-leg Fe–Ti joint angle during optogenetic stimulation of BRK (**a**) or FG (**b**) in tethered walking flies (each row is one example fly) grouped by stimulation onset mid-swing (left) or mid-stance (right). Swing phase indicated by grey background **c**, Probability (prob.) density of front-leg Fe–Ti joint angle if BRK or FG was stimulated mid-swing (left) or mid-stance (right). **d**, Average swing duration before and on mid-swing stimulation of BRK (left) or FG (right) for front legs (FL), mid-legs (ML) and hind legs (HL). *n* = 13–21 flies per genotype, multiple paired *t*-test. **e**, Averaged translational velocity during BRK + MDN (top) or BRK + BDN2 stimulation in decapitated flies (bottom). *n* = 7–11 flies per genotype, mean ± s.e.m., top red bar indicates optogenetic stimulation. **f**, 3D reconstructed leg joints during BRK + MDN or BRK + BDN2 stimulation. **g**, Fe–Ti joint angle values at swing initiation onset, median ± interquartile. **h**, Number of times per trial when front-leg Fe–Ti joint angle value entered the swing initiation zone (SIZ). **i**, Time spent with Fe–Ti joint angle in SIZ. **j**, Percentage of swings initiated after the front-leg Fe–Ti joint entered SIZ. **h**–**j** show median ± interquartile, Mann–Whitney test (***P < 0.001, **P < 0.01) (also Supplementary Video 5). **k**, Femoral muscle activity (1P) in Fe–Ti joint kept flexed (top) or extended (bottom) in control (left) or BRK>CsChrimson (right) flies. Top red bar indicates 1.6 s optogenetic stimulation. Mean ± s.e.m., *n* = 8–9 flies per group. **l**, Muscle activity (2P) in BRK>CsChrimson flies. Mean ± s.e.m., *n* = 7–8 flies per group. **m**, Femoral muscle anatomy with Fe–Ti flexed (top) or extended (bottom) in MHC-tau::GFP flies. Scale bars, 50 µm. **n**, Averaged trial ∆*F* images of two BRK>CsChrimson flies with Fe–Ti joint flexed (top) or extended (bottom) (Supplementary Video 6). Supplementary Table 1 shows full experimental genotypes and exact sample sizes. Source Data

The stepping cycle consists of a swing phase (leg in air) and stance phase (leg on ground). Given the strong ‘freezing-like’, BRK phenotype (Fig. 1g), we wondered whether BRK could cause the legs to ‘freeze’ at any point during a stepping cycle similar to a recent report in mice^2^. We found that in case of BRK mid-swing stimulation, the leg finishes the swing phase in an unaltered manner, but freezes as soon as it lands on the ground (Fig. 4a) when the femur–tibia (Fe–Ti) joint is in an extended position (Fig. 4c). Indeed, in these cases, swing duration is not altered when compared to prestimulus swing duration (Fig. 4d). However, for BRK mid-stance stimulation, the leg stays locked in place at the onset of the stimulation (Fig. 4a) leading to a broad Fe–Ti joint distribution across flies and trials (Fig. 4c). This is in contrast to FG stimulation in which the leg always assumes a relaxed stance position, regardless of when the optogenetic stimulus started (Fig. 4b,c). A similar result was obtained for other leg joints, suggesting it is not a Fe–Ti joint-specific phenomenon (Extended Data Fig. 6f and Methods).

This locking of joints during stance phase shows that the BRK phenotype is probably due to an active mechanism. We proposed that this could be driven by BRK VNC outputs upregulating the postural resistance reflex to lock the leg joints. The term ‘resistance reflex’ is used to describe postural reflexes in all legged animals that allow maintenance of posture while in a stationary state^36^. Upregulating such reflexes could lead to locking of leg joints while in stance phase as seen on BRK stimulation.

To test this hypothesis, we activated either MDN or MDN + BRK in decapitated flies and analysed their leg kinematics. As reported earlier^32^, decapitated flies showed backward walking on MDN activation. However, decapitated MDN + BRK stimulated flies remained stationary (Fig. 4e). This confirms that a VNC-specific circuit is recruited by BRK to arrest leg movements. During backward walking, the Fe–Ti joint of the front leg is extended before a swing is initiated (Fig. 4f,g). We therefore refer to Fe–Ti angles above 107° (Methods) as the ‘swing initiation zone’ (SIZ). On MDN + BRK stimulation, the Fe–Ti angle remained locked in place, and in most cases never reached the SIZ (Fig. 4h). In the few cases when it reached the SIZ (Fig. 4f), this rarely led to levation of the leg. The leg, despite being in an extended Fe–Ti position still remained firmly perched on the substrate as indicated by increased dwell time in SIZ (Fig. 4i) and decreased swing events in SIZ (Fig. 4j and Supplementary Video 5).

We performed a complementary analysis for forward walking while activating BDN2 or BDN2 + BRK in decapitated flies. In contrast to backward walking, during forward walking the SIZ for front-leg Fe–Ti lies in flexed angle values (Fig. 4g, SIZ is Fe–Ti less than 68°, Methods). Despite this difference, coactivation with BRK interrupted forward walking (Fig. 4e and Extended Data Fig. 6g,h) in a similar manner to that seen for backward walking (Fig. 4f–j and Supplementary Video 5). Taken together, this showed that BRK activation led the flies to resist both flexion and extension at the Fe–Ti joint.

**Extended Data Fig. 7:**
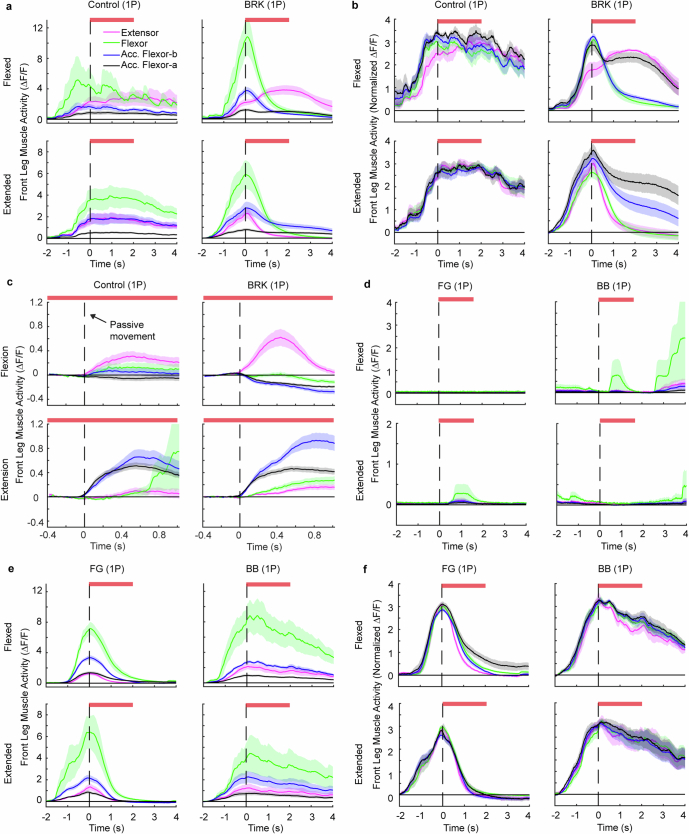
Different halt neurons have distinct effects on muscle activity. **a**. Left front leg femoral muscle activity (MHC>GCaMP6f, color coding as in Fig. 4k) measured under epifluorescence (1 P) while Fe-Ti joint is restrained in a flexed (top) or extended (bottom) position in control (left) and BRK-activated flies (right), with optogenetic stimulation (red bar) delivered during high baseline muscle activity. Light stimulation starts at vertical stippled line in velocity plots. n = 5-9 flies per group (flexed or extended), mean ± s.e.m. **b**. Same dataset as in (**a**), but plotted as variance-normalized ΔF/F for better visualization of change in accessory flexors and extensors (i.e. the high ΔF/F pre-stimulus values of flexors in (**a**) expand the Y-Axis). n = 5-9 flies per group (flexed or extended), mean ± s.e.m. **c**. Left front leg femoral muscle activity measured under epifluorescence (1 P) while the Fe-Ti joint is forcibly flexed (top) or extended (bottom) in control (left) or BRK-activated flies (right) during light stimulation (red bar). 1 s passive flexion/extension start at vertical stippled lines in plots. n = 32-40 trials pooled across 5 flies per genotype, mean ± s.e.m. **d**. Femoral muscle activity (1 P) in Fe-Ti joint kept flexed (top) or extended (bottom) in FG- (left) or BB-activated flies (right). Top red bar indicates 1.6 s optogenetic stimulation. mean ± s.e.m., n = 6-7 flies per group (flexed or extended). **e,f**. As in (**a,b**), but for FG (left) and BB activation (right) upon high baseline muscle activity, with calcium traces shown as ΔF/F (**e**) and variance-normalized ΔF/F (**f**), n = 6-7 flies per group (flexed or extended). Supplementary Table 1 shows full genotypes and sample sizes. Source Data

Resisting such movements, would require actively engaging specific leg muscles. We, therefore activated BRK and monitored front-leg femoral muscle activity with the Fe–Ti joint restrained in a flexed or extended position (Methods). In a flexed Fe–Ti position, activating BRK recruited accessory flexor-a and extensor. However, in an extended Fe–Ti position, BRK stimulation recruited accessory flexor-a and accessory flexor-b (Fig. 4k–n and Supplementary Video 6). Based on the literature^37,38^, we inferred that accessory flexor-a corresponds to the muscles innervated by slow motor neurons (also referred to as ‘reductors’) and is also implicated in maintaining joint position^38,39^. The position of accessory flexor-b indicates they are probably innervated by intermediate and/or fast flexor motor neurons that drive flexion movement that would resist forced extension. Extensors on the other hand, drive Fe–Ti extension and would therefore resist forced flexion. Thus, the muscle activity profile driven by BRK resembles a resistance-like state in each Fe–Ti position. Even in cases when there was spontaneous, prestimulus muscle activity, BRK drove a concerted change including inhibition of highly active muscles and activation of specific muscles to reach this same resistance state-like activity profile (Extended Data Fig. 7a,b and Supplementary Video 7).

To confirm this represents a resistance reflex-like activity, we passively flexed or extended the Fe–Ti joint and induced resistance reflex while monitoring muscle activity (Methods). Here, both control flies and BRK stimulated flies resisted these passive movements and showed a similar recruitment of muscles to that described above, with BRK stimulated flies showing a more robust response (Extended Data Fig. 7c). Taken together, this suggests that BRK drives a resistance-like state in the femoral muscles.

FG or BB activation did not induce any robust muscle activity in similar experiments (Extended Data Fig. 7d). Only in cases where there was prestimulus spontaneous muscle activity did FG lead to gradual inhibition of this activity (Extended Data Fig. 7e,f). We reasoned this could be because FG inhibits DNs driving the prestimulus spontaneous activity.

Combining the kinematics and muscle-imaging results, we conclude that BRK drives a stance phase-specific resistance state at the leg joints by means of a VNC-specific neural circuit.

## FG and BB are required during foraging

**Fig. 5:**
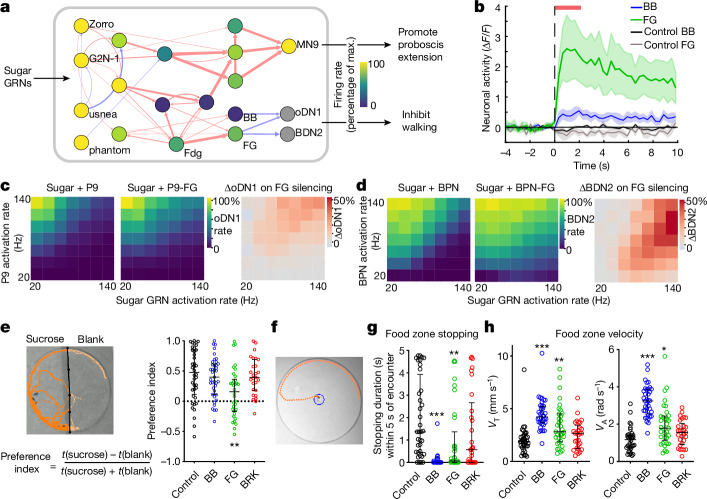
FG and BB recruited for halting in the context of feeding. **a**, In silico stimulation of sugar GRNs showing recruitment of FG and BB by sucrose sensory-motor pathway. Nodes are colour-coded by firing rate, and arrows weighted by synapse number as in Fig. 3. **b**, FG or BB activity (in vivo GCaMP7b imaging, mean ± s.e.m.) during 2 s of optogenetic stimulation (top red bar) of sugar GRNs (Gr5a), in starved flies. *n* = 4–9 flies per genotype. **c**,**d**, Simulation of oDN1 (**c**) or BDN2 (**d**) firing rate while coactivating P9 (**c**) or BPN (**d**) with sugar GRNs across range of stimulation rates in intact model (left) or with FG silenced model (middle). Difference between middle and left panels in both **c** and **d**, is depicted in respective right panels. **e**, Example fly trajectory from two-choice assay (left) and preference index (right) of sucrose over blank, for flies with BB, FG or BRK silenced (GtACR1). *n* = 27–40 flies per genotype, median ± interquartile, Kruskal–Wallis test followed by Dunn’s comparison with control (**P < 0.01). **f**. Example fly trajectory in the food-blob interaction assay showing the food-interaction zone (blue). **g**, Stopping-bout duration within 5 s of food encounter for flies with halt neurons silenced (GtACR1). **h**, Translational (left, *V*_T_) or angular (right, *V*_A_) velocity of flies in the food-zone within 5 s of food encounter for flies in **g**. **g**,**h**, *n* = 29–36 flies per genotype, median ± interquartile, Kruskal–Wallis test followed by Dunn’s comparison with control (***P < 0.001, **P < 0.01, *P < 0.05). Supplementary Video 8. Supplementary Table 1 shows full experimental genotypes and exact sample sizes. Source Data

**Extended Data Fig. 8:**
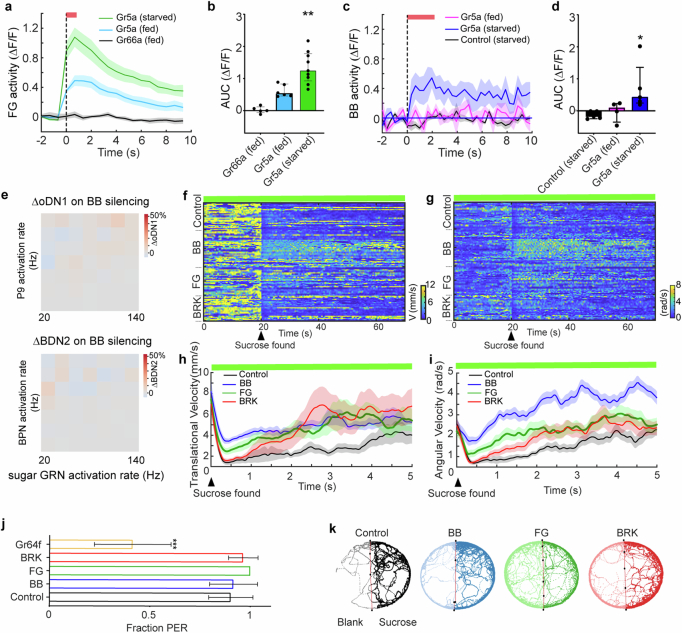
Evaluating the role of different halt neurons in the context of feeding. **a**. FG activity (GCaMP7b) during activation (ChrimsonR) of sweet gustatory sensory neurons (Gr5a, in fed or starved flies) or bitter gustatory sensory neurons as controls (Gr66a, in fed flies). 2 s optogenetic activation (red bar) starts at vertical stipple line. n = 5-9 flies per genotype, mean ± s.e.m. **b**. Area under the curve (AUC) for 0-1 s after stimulation onset in each group in (**a**), median ± interquartile, dots indicate number of flies, Kruskal-Wallis test followed by Dunn’s comparison with control (**p < 0.01). **c**. BB activity (GCaMP7b) with Gr5a activated (in fed or starved flies) or without Gr5a neurons activated (fed flies). 2 s optogenetic activation (red bar) starts at vertical stipple line. n = 4-5 flies per genotype, mean ± s.e.m. Traces from starved flies (Gr5a and control groups) are also plotted in Fig. 5b. **d**. Area under the curve (AUC) for 0-2 s after stimulation onset in each group from (**c**), median ± interquartile, dots indicate number of flies, Kruskal-Wallis test followed by Dunn’s comparison with control (*p < 0.05). **e**. Top: change in activity of oDN1 while co-stimulating P9 and sugar GRNs across a range of stimulation rates in model with BB silenced. Bottom: change in activity of BDN2 while co-stimulating BPN and sugar GRNs across a range of stimulation rates in model with BB silenced. Normalization of firing rate as in model with FG silencing in Fig. 5c, d. **f, g**. Translational velocity (**f**) and angular velocity (**g**) heatmaps of starved, free-walking flies with BB, FG and BRK silenced (GtACR1), compared to control flies. Green bar on top indicates green light stimulation. Arrowheads indicate the time point when the fly first encounters sucrose (arrowheads). n = 29-36 flies per genotype. **h,i**. Data from (**f, g**) showing translational velocity (**h**, mean ± s.e.m.) and angular velocity (**i**, mean ± s.e.m.) during the 5 s post-food encounter. **j**. Proboscis extension response (PER) upon sucrose presentation in flies with FG, BB, BRK, or Gr64f (sugar sensory neurons used as positive controls) silenced (GtACR1) compared to control flies. n = 26-34 flies per genotype, mean with 95%CI, Fisher’s exact test for comparison with control (***p < 0.001). Also see Supplementary Video 8. **k**. Overlay of trajectories of 3 flies in the two-choice assay for BB, FG or BRK silenced flies (GtACR1), compared to controls. Red line demarcates the blank side from the sucrose side. Supplementary Table 1 shows full genotypes and exact sample sizes. Source Data

We next addressed if and when the animals use the FG, BB and BRK halting pathways. Mining the connectome for inputs to the halting neurons revealed that FG and BB are downstream to the sugar sensory pathway, and directly connected to Fdg neurons previously shown to drive halting and proboscis extension for feeding^40,41^ (Fig. 5a). We therefore activated sugar gustatory receptor neurons (GRNs) both in silico and in vivo. In both cases, FG was strongly recruited whereas BB showed weak and variable activity. (Fig. 5a,b). Moreover, in vivo, FG showed stronger responses in starved flies compared to fed flies (Extended Data Fig. 8a,b), whereas BB only responded (weakly) in starved flies (Extended Data Fig. 8c,d). These state-dependent responses could be driven by differences in the feeding drive due to hunger signals impinging on the sugar sensory pathway^41^. To understand how different feeding and walking drives might converge on the previously implicated walking-promotion nodes (oDN1 and BDN2), we coactivated sugar GRNs with either P9 or BPN walk neurons at varying activity levels, and monitored oDN1 and BDN2 firing rates, respectively. Both oDN1 and BDN2 were tightly regulated as a function of walking and feeding pathway stimulation, showing that there is an excitation–inhibition balance at these nodes (Fig. 5c,d). To test whether FG is critical for relaying the inhibition from sugar GRNs to the walking nodes, we repeated the above simulation, but while silencing FG in the model. This clearly shifted the excitation–inhibition balance at the walking nodes, such that we could now see release of inhibition on oDN1 and BDN2 when compared to the results in the FG intact model (Fig. 5c,d). This suggests that FG may be relevant for halting in the context of foraging and feeding. Because BB is only weakly recruited in our model, we did not see big changes at BDN2 or oDN1 when we repeated the simulation with BB silenced (Extended Data Fig. 8e).

To confirm the role of halt neurons during feeding, we optogenetically silenced these neurons, while providing starved flies with a choice between blank substrate (dried filter paper) versus sucrose substrate (dried filter paper + sucrose, where flies can sense the sucrose but cannot consume it^42^). Control flies or flies with BRK or BB silenced spent most of their time on the sucrose side, whereas FG silenced flies were unable to preferentially stay on the sucrose side (Fig. 5e and Extended Data Fig. 8k), confirming the importance of FG for stopping on sugar detection. At the start of this assay, flies were randomly distributed on the sucrose and blank side making it difficult to analyse locomotor changes when the fly first encountered the sucrose. To analyse these first encounters, we quantified interaction of flies with a small food-blob, placed at the centre of a large circular arena (Fig. 5f). All flies slowed down when they first encountered the food-blob, probably because, unlike the previous assay, here they could detect the food-blob by textural changes and consume it. However, FG- and BB-silenced flies showed shorter stopping bouts and increased velocities near the blob compared to controls (Fig. 5g–h and Extended Data Fig. 8f–i). Specifically, BB-silenced flies were indistinguishable from controls before food encounter, but showed prolonged increase in both translational and angular velocities after food encounter (Extended Data Fig. 8f–i). This could show a potential role for BB in constraining forward and angular velocities during foraging-type behaviours that involve a local search following food encounter^43,44^. Such post food-encounter behavioural state transitions have been well described in flies^43,44^. However, these are not accounted for in our computational model, or in the functional imaging experiments (fly tethered vertically and cannot walk, Methods). This could explain the weaker BB responses observed on in vivo or in silico sugar-GRN stimulation.

Taken together, this confirms that FG and BB are recruited for halting and locomotor control in the context of feeding and foraging. Notably, connectomics revealed that this halting pathway diverges from the previously described proboscis extension pathway^7,41^ (Fig. 5a). In agreement with this, FG or BB silencing had no effect on the proboscis extension response (PER) when tethered flies were presented with sucrose (Extended Data Fig. 8j).

## BRK is required during grooming

**Fig. 6:**
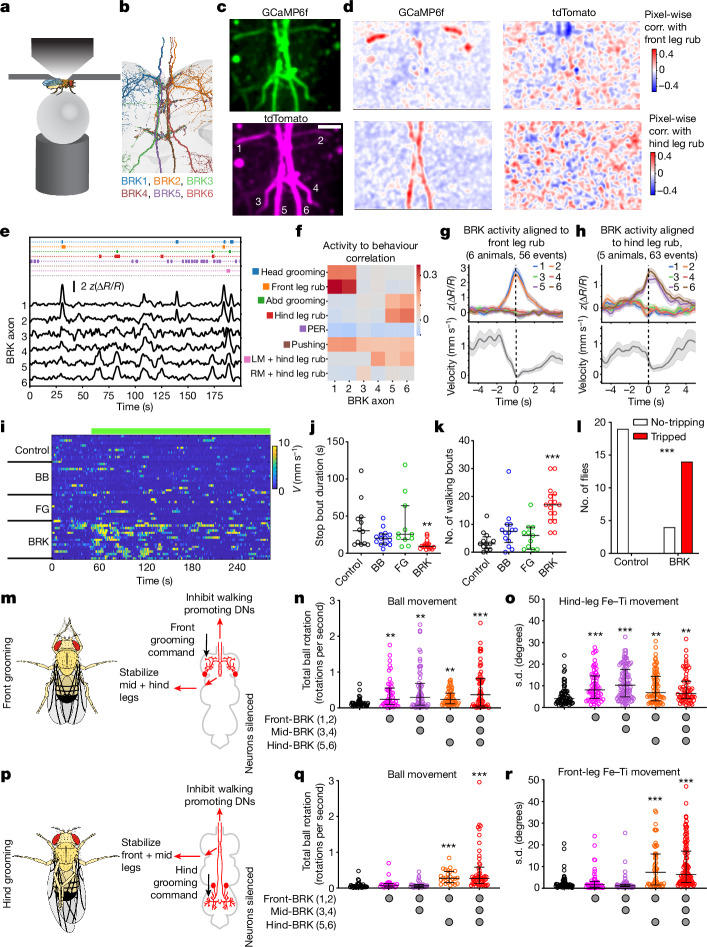
BRK is recruited for halting during grooming. **a**, Schematic of in vivo VNC imaging. **b**, Electron microscopy segmentation of BRK neurons near the output zone in VNC tectulum region. **c**, Averaged image of green (GCaMP6f) and magenta (tdTomato) channels with BRK neuron 1–6 juxtaposed to their respective axons (1, 2, front-leg BRK; 3, 4, mid-leg BRK; 5, 6, hind-leg BRK). **d**, Pixel-wise correlation map from **c**, showing pixels with intensity correlated to front-leg rubbing events (top) or hind-leg rubbing events (bottom). **e**, Imaging session showing all ethograms (top) and *z*-scored ∆*R*/*R* (bottom) for each BRK neuron. **f**, Correlation (corr.) between BRK activity and annotated behaviours (data pooled across six flies; Abd, abdomen; PER, proboscis extension response). **g**,**h**, *z*-scored ∆*R*/*R* (top) aligned with respect to initiation of front-leg rubs (**g**) or hind-leg rubs (**h**), and corresponding forward velocity of the fly (bottom). Colour code as in **b** (also Supplementary Video 9). **i**–**k**, Translational velocity heatmap (**i**), stop bout duration (**j**) and number of walking bouts (**k**) of grooming flies with BB, FG or BRK silenced (GtACR1; top green bar in (**i**) depicts optogenetic silencing), *n* = 11–16 flies per group, median ± interquartile, Kruskal–Wallis test followed by Dunn’s comparison (***P < 0.001, **P < 0.01). **l**, Number of flies that tripped versus remained stable while grooming in control or BRK silenced flies (GtACR1), *n* = 18–19 flies per group, Fisher’s exact test (***P < 0.001) (also Supplementary Video 10). **m**, Proposed role of BRK1-2 in stabilizing the mid- and hind legs during front grooming. **n**,**o**, Ball movement (**n**) and standard deviation of Fe–Ti flexion angle of the hind legs (**o**) during front grooming in decapitated flies, with subsets of silenced BRK neurons (GtACR1). *n* = 46–60 bouts, median ± interquartile, Kruskal–Wallis test followed by Dunn’s comparison (***P < 0.001, **P < 0.01).  **p**, Proposed role of BRK 5-6 in stabilizing the front- and mid-legs during hind grooming. **q**,**r**, Ball movement (**q**) and standard deviation of Fe–Ti flexion angle of the front legs (**r**) during hind grooming in decapitated flies, with subsets of silenced BRK neurons (GtACR1). *n* = 35–73 bouts, median ± interquartile, Kruskal–Wallis test followed by Dunn’s comparison (***P < 0.001). (See also Supplementary Video 11). Supplementary Table 1 shows full experimental genotypes and exact sample sizes. Source Data

To understand when flies recruit BRK neurons, we monitored in vivo BRK activity in spontaneous walking and halting flies (Methods and Fig. 6a). We imaged a region of the VNC that permits identification of the axons belonging to each of the six BRK neurons (Fig. 6b,c). We noticed that BRK is recruited only during a subset of halting events. By annotating synchronously recorded behaviour we found that BRK activity correlated with either ‘ball-pushing’ or ‘grooming’ events (Fig. 6d–f). As ball-pushing is probably a special behaviour that only happens in a tethered fly walking on an air-supported ball, we focused on the grooming events. BRK activity correlated in a segment-specific manner to the respective grooming events (Fig. 6f). Most prominent among these were front-leg rubs and hind-leg rubs that coincided with large correlated activity bumps in front and hind BRK, respectively (Fig. 6d–f and Supplementary Video 9). Aligning BRK activity profile across multiple flies and grooming events, showed that the segment-specific BRK activity starts ramping up before the grooming event and synchronized with a coincident dip in walking velocity (Fig. 6g,h).

**Extended Data Fig. 9:**
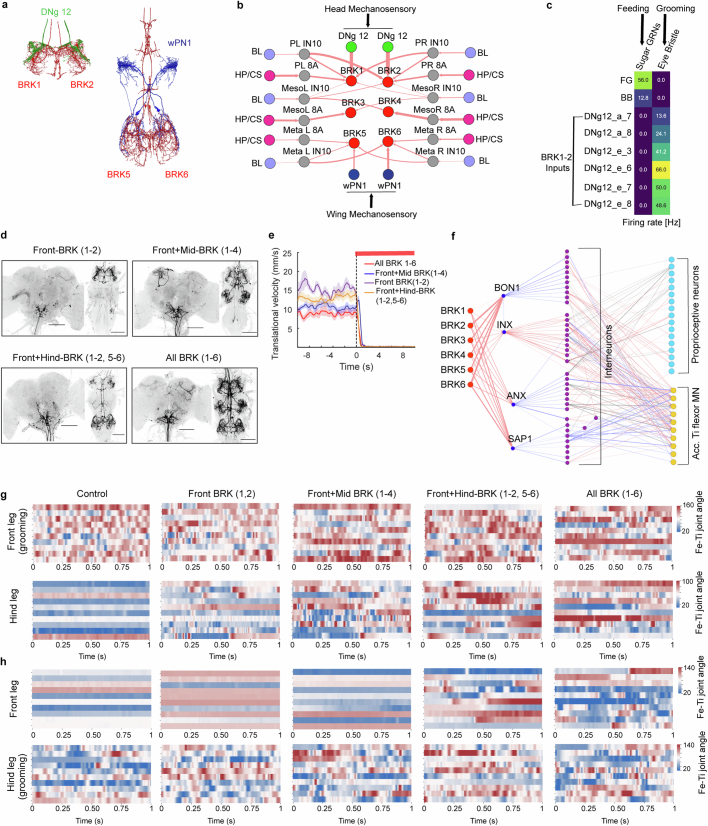
Connectome and behavior-based segment-specific analysis of BRK neurons. **a**. EM visualization of head-grooming command neurons DNg12 that connect with BRK1-2 with 674 synapses (left) and wing-grooming command neurons wPN1 that connect with BRK5-6 with 284 synapses (right). **b**. Connectivity diagram showing segment-specific grooming command inputs to BRK from head-grooming pathway (DNg12), wing-grooming pathway (wpN1) or potential leg-grooming pathway. (BL: Bristle, HP: hairplate, CS: Campaniform sensilla). **c**. Simulation of feeding or grooming contexts by modeling sugar GRNs and eye bristles activation in the connectome-constrained network model, respectively. Colors and associated values in heatmap indicate firing rate (Hz) of FG, BB and grooming command DNs (DNg12s), which project to the front-BRK 1-2 neurons. **d**. Immunohistochemistry images of the genetic drivers used in Fig. 6n–r, showing targeting of segment-specific BRK neurons in the brain and VNC. (GTACR1-EYFP - expression; scale bars: 100 μm). **e**. Trial averaged translational velocity (mean ± s.e.m.) of free-walking flies with CsChrimson expressed only in BRK1-2, BRK1-4, BRK1-2,5-6 or BRK 1-6. 10 s optogenetic stimulation (red bars) starts at vertical stippled lines in velocity plots. n = 5–16 flies per genotype. **f**. 2^nd^ order outputs of BRK in the VNC receive input from sensory neurons (mostly proprioceptive, blue) and provide output to accessory tibia flexor motor neurons (Acc. Ti flexor MN, yellow). In (**b,f**) arrow thickness indicates synaptic strength (5-400 synapses, 5-300 synapse resp.). Red indicates excitatory connections; blue indicates predicted inhibitory connections. **g**. Examples of femur-tibia flexion angle heatmaps for front (top) and hind (bottom) legs in tethered, decapitated flies where front-grooming was induced, while silencing different subsets of BRK neurons. Quantified in Fig. 6o. **h**. As in (**g**), for tethered, decapitated flies where hind-grooming was induced. Quantified in Fig. 6r. Also see Supplementary Video 11. Supplementary Table 1 shows full genotypes and exact sample sizes. Source Data

Fittingly, in the connectome, we found that DNg12, a command neuron that drives head-grooming with front legs^45^ provides most input to front BRK (BRK1-2), whereas wPN1, a command neuron that drives wing grooming with hind legs^46^ provides most input to hind BRK (BRK5-6, Extended Data Figs. 5a and 9a–c). We also identified potential leg-grooming sensory-motor pathways from each leg to corresponding segment BRK neurons (Extended Data Fig. 9b). Thus, BRK neurons receive distinct segment-specific grooming command inputs. However, all BRK share common outputs (Extended Data Fig. 2b) that lead to halting with upregulation of postural reflexes (Fig. 4). What could be the role of the BRK outputs during grooming?

During grooming, flies halt and lift a subset of legs (usually two legs) while supporting their body weight on the remaining stationary legs. We wondered whether BRK was necessary for both halting and stability during grooming. We therefore optogenetically silenced BRK in flies that were induced to groom by powdering them^47,48^. BRK silencing did not abolish grooming, but led to interruption of continuous grooming by intermittent walking events (Fig. 6i–k) confirming BRK’s role for halting during grooming. Control flies or flies with FG or BB silenced showed prolonged uninterrupted grooming (Fig. 6i–k). Moreover, we found that BRK silenced flies become destabilized during grooming (Supplementary Video 10). To address if this destabilization was due to the VNC outputs of BRK, we repeated this experiment in decapitated flies. Consistent with the literature^48^, control decapitated flies showed robust grooming when covered with powder. Because decapitated flies do not have an inherent forward walking drive like intact flies, it was unsurprising that we did not see any walking on BRK silencing. However, we saw that powdered, decapitated BRK silenced flies often lost their balance and tripped while grooming, confirming BRK’s role in stabilization during grooming (Fig. 6l and Supplementary Video 10).

On the basis of the above observations, and given that BRK output only affects legs in stance, we proposed that BRK is recruited by segment-specific grooming commands to halt and stabilize the posture of the stationary (non-grooming) legs used for balancing the body weight. For instance, during front grooming, command neurons (DNg12s) would recruit the front BRKs that in turn stabilize mid and hind legs (Fig. 6m), whereas during hind grooming, the corresponding grooming commands (wPN1) would recruit hind-BRKs that in turn stabilize the front and mid-legs (Fig. 6p).

To test this segment-specific role of BRK, we designed an assay in which we silenced BRK subsets, while inducing segment-specific grooming. We generated split-Gal4 lines that targeted front BRK (BRK1-2), front + mid-BRK (BRK1-4) or front + hind BRK (BRK1-2, BRK-5-6), and confirmed that activating these BRK subsets drives halting in all cases (Extended Data Fig. 9d,e). Next, we optogenetically silenced these BRK subsets and induced front or hind grooming using a powdered brush in tethered decapitated flies placed on an air-supported ball (Supplementary Video 11). We performed leg kinematics analysis of front and hind legs (Methods). As expected, control flies were stable during the grooming events as indicated by low ball movement and low Fe–Ti movement of the non-grooming legs (Fig. 6n–r and Extended Data Fig. 9g–h). Conversely, when front-BRK silenced flies were induced to perform front grooming, they were destabilized as shown by increased ball movement (Fig. 6n) and hind-leg movements (Fig. 6o and Extended Data Fig. 9g). However, if flies with silenced front BRK but intact hind BRK (front-BRK>GtACR1 or front + mid-BRK>GtACR1), were induced to perform hind grooming, they showed control-like behaviour and groomed hind legs while stabilizing the front legs (Fig. 6n–o and Extended Data Fig. 9h). On the other hand, flies with hind BRK silenced (front + hind BRK>GtACR1 or all-BRK>GtACR1), were destabilized during hind grooming bouts (Fig. 6q,r and Extended Data Fig. 9h).

Taken together, this shows that BRK is recruited in a segment-specific manner for halting and stability in the context of grooming. We thus explain distinct contexts in which each halting pathway is recruited. Consistent with this, activating head-bristle sensory projections (grooming context) in the brain connectome model, drove strong activity in DNg12s (BRK inputs) but not in FG or BB, whereas activating sugar GRNs (feeding context) recruited FG and BB but not DNg12s (BRK inputs) (Extended Data Fig. 9c).

## Connectomics predicts halt neurons

**Extended Data Fig. 10:**
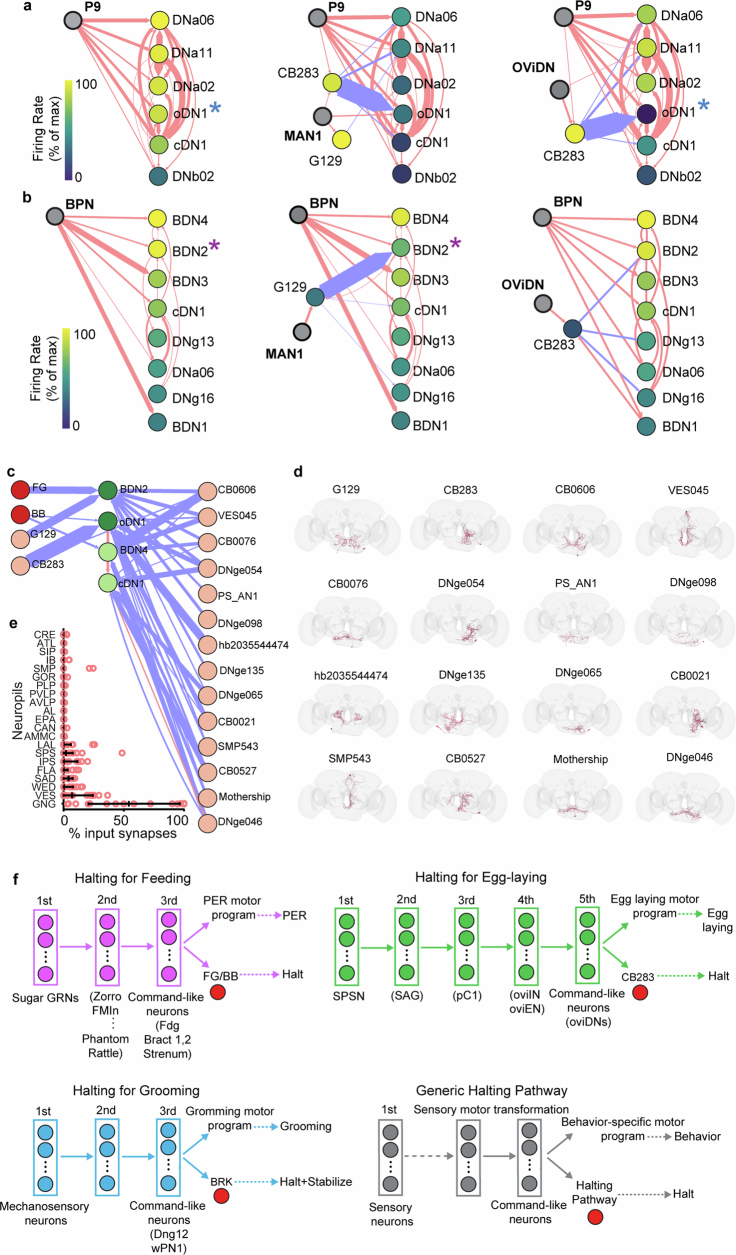
Connectome-guided prediction of novel halting pathways. **a,b**. Simulated firing rate for activating P9, P9 + MAN1, P9+OViDN (**a**) and BPN, BPN + MAN1, BPN+OViDN (**b**). Nodes in the wiring diagram are color-coded based on firing rate in the full simulation, identical to the heatmap; color-scale is normalized to the maximum trial averaged firing rate achieved in the case of the respective walk neuron activation simulation. Arrow thickness indicates synaptic strength (5-400 synapses). Red indicates excitatory connections; blue indicates inhibitory connections. Asterisks indicate neurons (oDN1, BDN2) selected for further analysis in Fig. 3. **c**. Connectome based prediction of halting neurons. Dark red filled circles indicate empirically verified halt neurons and light red filled circles indicate predicted halt neurons. Dark green filled circles indicate empirically verified walk neurons and light green filled circles indicate predicted walk neurons. Arrow thickness indicates synaptic strength (i.e. synaptic counts). Red indicates excitatory connections; blue indicates predicted inhibitory connections. **d**. EM segmentation of predicted halt neurons in the brain. **e**. Input brain region for predicted stop neurons. **f**. Sensory-motor circuit layout for stationary behaviors including feeding, egg-laying, grooming and for a generic halting pathway (grey). Distinct sensory pathways recruit specific command-like neurons – such as Fdg for feeding (magenta), oviDNs for egg-laying (green) and DNg12/wPN1 for grooming (blue) – that initiate context appropriate behaviors, by recruiting context-specific halting pathways (red) and motor programs. Filled circles represent neurons; solid arrows indicate monosynaptic connections; dotted arrows indicate indirect connections. Source Data

Having identified the critical nodes in the walking pathways that are targeted by halt neurons, we aimed to gain insight into other halting pathways that could be relevant in other contexts. Although we could not identify some previously described slowing^4^ or halting^5^ neurons in the connectome, we could locate two neurons that were implicated indirectly in promoting halting, namely MAN (assists backward walking by inhibiting forward walking^31^) and oviDN (command neuron for egg-laying^49^ that also induces halting^50^). In similar simulations as in Fig. 3, both MAN and oviDN, through predicted GABAergic neurons (G129, CB283), suppressed walking-promotion neurons including oDN1 and BDN2 (Extended Data Fig. 10a,b). This showed other routes in addition to FG, BB, BRK that could inhibit walking-promotion nodes. To find all such routes, we generated a list of predicted halt neurons (Extended Data Fig. 10c,d) as those with strong inhibitory inputs to oDN1 and BDN2, most of which also inhibit other potential walking-promotion DNs (for example, cDN1, BDN4). As these neurons will ultimately control the excitation–inhibition balance at the walking-promotion nodes, these could also be involved in velocity control (‘slowing down’) and not just halting. These predicted halt neurons receive input from different upstream neurons, and could underlie halting in other behavioural contexts (fear, sleep, courtship and so on).

## Discussion

Our work explains two fundamental halting mechanisms in *Drosophila*. The first mechanism, categorized as the walk-OFF mechanism causes halting by inhibition of walking-promotion neurons. This could be compared to taking one’s foot off the gas pedal of a car, and is analogous to action-suppression mechanisms proposed in different behaviours including walking, across organisms^11,51–56^. In this connectomics-driven work, we could go beyond identification of this mechanism and gain major insights into the organization of the halting and walking-control circuits in flies. We found that (1) walking initiation neurons (P9, BPN, MDN) recruit populations of heterogeneous DNs by means of SEZ outputs that are poised to recruit multiple downstream VNC circuits for initiating specific walking patterns, (2) halting and walking pathways converge at specific nodes in these DN populations (for example, oDN1, BDN2) whose excitation–inhibition balance probably dictates the walking state and (3) different halting pathways inhibit different subsets of walking-promotion DNs. Specifically, turning and forward-walking-promoting DNs could be differentially inhibited by BB and FG, respectively. This shows a modularity in the descending control of turning and forward walking that is consistent with recent results from neuronal population imaging and modelling work in *Drosophila*^12,34,57^. This also aligns with modularity at the level of brainstem spinal projection neurons observed in vertebrates (zebrafish^58^, mice^6,18,59^), indicating that vertebrates could also have halting pathways that differentially inhibit forward locomotion and turning.

The second mechanism, categorized as the brake mechanism, drives active resistance at the leg joints in the legs that are on the ground (stance phase), much like the brakes of a car resisting motion of the wheels. Also, similarly to how one releases the accelerator pedal while pressing on the brakes to avoid skidding, the newly identified BRK neurons inhibit the walking-promotion DNs through their brain outputs while simultaneously promoting resistance in the legs through their VNC outputs. The pathway from BRK to leg muscles is probably gated by proprioceptive inputs that confer the stance phase-specific behavioural response (Extended Data Fig. 9f). This BRK mechanism is consistent with their recruitment during grooming when the fly needs to both inhibit walking and balance its body on a subset of stationary legs. A similar mechanism could be relevant in other halting contexts in flies (for example, when flies lift their legs for lunging during aggression) or other animals for execution of halting with increased stability. Recent work in mice has uncovered a brainstem neuronal population (Chx10-Gi) that drives halting with legs in stance^1,59^. However, these neurons were not shown to drive upregulation of postural reflexes as seen for BRK. Moreover, recent work shows subsets of Chx10-Gi neurons being relevant in different aspects of walking, stopping, turning or grooming^18,59,60^. Our results encourage the search for BRK-like mechanism in these Chx10-Gi subsets or other BRK-analogous spinal ascending pathways in vertebrates.

Comparing across connectome-informed circuits of all verified or predicted halting pathways in this study, we isolated a generic neural circuit motif for halting. For promoting halting to allow stationary behaviours such as feeding, egg-laying or grooming, the halting pathway branches off from the behaviour-specific sensory-motor pathway and impinges on walking-promoting circuits (Extended Data Fig. 10f). The halt neurons themselves need not encode for the behavioural-specific motor output (for example, proboscis extension, ovipositor extrusion or grooming movements) but rather drive the initiation and maintenance of a halted state during the behaviour. This halting branch is typically located downstream to command-like neurons in the sensory-motor circuit (note that predicted halt neurons also receive their inputs primarily in the SEZ region, which is closer to the output of the brain, Extended Data Fig. 10e,f). This emergence of modularity downstream in the circuit could be an efficient circuit-design for targeting similar population of walking-promotion neurons in different contexts.

Despite this conserved motif shared across all halting pathways, the specific targets and mechanisms deployed by the halt neurons are distinct. This distinction may confer the specificity to how halting versus walking decisions are executed in a context-appropriate manner.

## Methods

### Experimental animals

We used *Drosophila melanogaster* raised on standard cornmeal-agar medium supplemented with baker’s yeast and incubated at 25 °C with 60% humidity and a 12 h light–dark cycle throughout development and adulthood unless otherwise stated. The optogenetic manipulation experimental flies were collected on retinal food and again transferred to fresh retinal food 1–2 days before testing. Retinal food contains standard fly food with freshly added 400 μM all-*trans* retinal (Sigma-Aldrich, catalogue no. R2500). These flies were kept in the dark for their entire life-cycle until test. Age and sex of animals tested is indicated in the sections below. All full experimental genotypes, exact sample size per genotype and source of the genetic reagents are described in Supplementary Tables 1 and 2.

### Identification and generation of halt neuron specific drivers

The neural-activation screen was composed of an extended version of our previously published work^19^ with added lines from the SEZ-split-Gal4 collection^20^. Whereas in our previous work^19^ we focused on lines that increased walking on optogenetic stimulation, here we focused on lines that decreased walking. We could narrow the set of interesting lines to 11 drivers based on their locomotor phenotype and expression levels. Among the SEZ lines, we focused on three lines (SS40909, SS31326, SS31328) in which we could unambiguously identify the neurons as FG and BB both in the light microscopy^24^ as well as electron microscopy^8,28,29,61^ datasets. Additionally, we found three Gal4 drivers that drove the strongest halting phenotypes (R37F06, R36G02, VT12408). By comparing available or generated stochastic labelling images (we performed this for VT012408 as described in ref. ^19^, MCFO images were available for R36G02 and R37F06, ref. ^24^), we identified that these three lines labelled BRK neurons. To validate this further, we devised a split-Gal4 screen with R36G02-Gal4DBD and candidate BRK targeting p65-ADs identified by using the NeuronBridge toolkit^24^. This (Extended Data Fig. 1d) helped generate split-Gal4 drivers for labelling BRK neurons (Fig. 1).

### Identification of neurons in connectome

We used image database search tools^24,25^ and co-ordinate transform tools^23,26^ to identify neurons across light microscopy and electron microscopy datasets. Maximum intensity colour-depth images from light microscopy and electron microscopy for each neuron used in this study are shown in Extended Data Fig. 5a. Electron microscopy identifiers of all neurons from this work are detailed in Supplementary Table 3. Further details of comprehensive DN and AN proofreading and identification in the FlyWire and FANC datasets are now reported in ref. ^62^.

### Optogenetic activation in untethered animals

All optogenetic activation experiments in untethered animals were performed using 6–9-day-old female flies. The flies were loaded in behavioural arenas as described in ref. ^19^ (44 mm bowl-shaped arena made of 1.5% agarose gel). The videos were recorded as in ref. ^19^ using a FLIR BlackFly-S camera (FL3-U3-13Y3M-C) at a resolution of 1,280 × 1,024, at 30 Hz. The camera was fitted with an adjustable focus lens (LMVZ990-IR) and near infrared bandpass filter (Midopt BP850) to allow infrared imaging without artefacts from visible light. A custom designed light-emitting diode (LED) panel^19^ provided backlit illumination with infrared (850 nm), green (530 nm) or red (630 nm) light. Both intensity and pulsing of each wavelength could be independently controlled and synchronized to the recording camera through transistor–transistor logic pulses generated using a custom Arduino circuit. The bowl-shaped arena was backlit with infrared light (850 nm) for video recording. We also provided continuous dim green light (0.0031 mW mm^−2^) during all optogenetic activation experiments to avoid a jumping response in flies due to sudden bright red light exposure. The green light level was adjusted as to not drive optogenetic stimulation even in a very sensitive reagent (MDN>CsChrimson). All experiments were performed at 25 °C unless stated otherwise. Videos were tracked using FlyTracker software^63^ and data were analysed in MATLAB.

#### Activation in free-walking flies

Experimental flies were loaded in the setup described above, and allowed to walk freely to be assayed for optogenetics induced halting (Figs. 1a–c and 2 and Extended Data Figs. 1, 2d–g, 4b,c and 9e). The light stimulation protocol consisted of red light pulsed at 50 Hz (5 ms pulse width, average intensity at arena surface of 0.038 mW mm^−2^) in a sequence of 50 s OFF/10 s ON, repeated five times. A trial was defined as 10 s OFF followed by 10 s ON for analysis.

#### Activation in powdered flies

Flies were covered with powder (Reactive Yellow 86, Santa Cruz Biotechnology, catalogue no. sc-296260) to induce grooming^19,48^. Powdered flies were loaded in bowl-shaped arenas described above, and assayed for walking initiation with red light intensity of 0.04 mW mm^−2^ pulsed at 100 Hz (Fig. 3j). The light stimulation protocol consisted of 50 s OFF/10 s ON sequence, repeated five times. For analysis, 10 s OFF followed by 10 s ON was considered as one trial.

#### Data analysis

FlyTracker^63^ output was used to quantify translational and angular velocities as in ref. ^19^. Angular velocity values correspond to ‘absolute angular velocity’. Rotation is defined as integral of angular velocity as in ref. ^19^. Pivots were defined as time periods with high angular velocity (more than two rotations per second) and low translational velocity (less than 5 mm s^−1^) after smoothing with 0.5 s window. These pivot thresholds are set based on empirical observation, as well as previous literature^64–66^ indicating values for slow walking and high turning. For coactivation experiments (Fig. 2), distance and rotation were calculated for the entire stimulation duration or just first 2 s of stimulation as indicated. In all experiments in Fig. 2 in which we compared coactivation of walk + halt neuron phenotypes, we restricted statistical analysis to a period of 2 s after stimulation onset. All walk activation phenotypes start declining after this 2 s mark. Moreover, in case of MDN activation, the flies start switching between backward and forward bouts after this 2 s mark. This 2 s period was thus selected to restrict the analysis to the clean and strong part of the walk phenotype. The results were identical if the time window was changed 2 ± 1 s.

### Optogenetic silencing in untethered animals

All optogenetic silencing experiments were performed using 6–9-day-old female flies (unless stated otherwise) in the same setup and tracking and analysis pipeline as the activation experiments. The 530 nm green LED used for silencing was adjusted to 0.0255 mW mm^−2^ average intensity at the arena walking surface to silence neurons expressing GtACR1.

#### Silencing in free-walking flies

Flies were loaded in bowl-shaped chambers kept at 30 °C (to elevate baseline walking) and assayed for decrease in walking velocity on silencing (Fig. 3k). The light stimulation protocol consisted of a 60 s OFF/30 s ON sequence, repeated three times, for a video duration of 5 min.

#### Silencing in powdered intact flies

Flies were powdered as described above and assayed for interruption of grooming (Fig. 6i). The green light stimulation protocol consisted of 60 s OFF followed by continuous ON for a duration of 6 min.

#### Silencing in powdered decapitated flies (assay for tripping quantification)

The flies were decapitated using forceps and the neck was sealed using ultraviolet-cured glue (Bondic). Flies that recovered well from this procedure (based on good self-righting and grooming behaviours) were chosen for experiments. The experimental flies were powdered (as mentioned above) and loaded in flat-floor arenas (50 mm diameter and 3 mm height described in ref. ^19^). The light stimulation protocol consisted of 30 s OFF/10 s ON sequence of green light, repeated three times, for a video duration of 3 min. The flies that lost balance and ended in an upside-down position (with all legs off the ground) in at least one out of three light ON periods were recorded as tripped (Fig. 6l and Supplementary Video 10).

#### Data analysis

Velocities, distance and rotation were quantified as stated above. Stopping events were defined as instances in which smoothed translational velocity (1.5 mm s^−1^) was below a threshold defined previously^31^. Tripping events were quantified manually.

### Feeding related neuronal silencing assays

Here, 1–3-day-old female flies were transferred to retinal food and allowed to feed for 4 days. Flies were then wet-starved with 0.4 mM retinal in water before testing to induce starvation.

#### Sugar preference assay

This assay was performed using previously described setup and conditions^42^. Here, 36-hour starved female flies were loaded in flat-floor circular behavioural arenas (50 mm diameter) described above. The circular arena floor was covered with two halves of semi-circular filter paper, which had been soaked with either water or 2 M sucrose and left to dry overnight. Flies were introduced into the chamber and allowed to explore and choose a preferred side for a duration of 4 min (Fig. 5e). Green light was provided throughout assay duration for GtACR1-based neuronal silencing. By the time video recording started, most flies had encountered both sides of the chamber, implying that at *t* = 0 flies are considered as having been exposed to sucrose. Video recording, tracking and analysis was performed as described above.

#### Sucrose-blob interaction assay

A 5 µl drop (3 mm diameter) of 1% agarose solution containing 200 mM sucrose was placed in the centre of the flat circular arena (50 mm diameter). One fly per arena was loaded and allowed to explore and find the sucrose drop. Green light was provided throughout the assay duration for GtACR1-based neuronal silencing as described above. Flies that encountered the sucrose before the video recording started were discarded from the analysis to ensure accurate capture of the first feeding bout. Video recording and tracking was performed as described above. A fly within 3 mm from the centre of the sucrose blob was considered as interacting with it (as both the sucrose blob and the fly are similar size; that is, roughly 3 mm). The food-zone was then defined as 6-mm-diameter circle centred around the sucrose blob (Fig. 5f). The exact frame corresponding to when the fly first found the sucrose was manually annotated, and the data were aligned to this time point (Extended Data Fig. 8f–i). Quantification of food-zone stopping and velocities was performed within 5 s of finding the sucrose. Stops were defined as smoothed-velocity less than 2 mm s^−1^ for ten frames (when flies stayed in one spot for long as happens when they feed, the tracker often induced a jitter that led to artificial velocity values, this definition helped extract true stopping events). For depicting velocity heatmap and averaged velocity plots depicting pre- and postencounter profile, we filtered the dataset to select cases that showed at least 20 s pre-encounter period and 50 s postencounter period. Most flies were still contained in this dataset.

#### PER assay

The PER assay in Extended Data Fig. 8j was performed as described in ref. ^41^. Data were analysed using Fisher’s exact test between test and control genotypes.

### High-resolution 3D leg kinematics analysis

#### Setup

The setup consisted of eight cameras (FLIR BFS-U3-16S2M-CS, fitted with InfiniStix 194100 lenses and near infrared bandpass filters (Midopt BP850)) surrounding a ball holder (Extended Data Fig. 3a), such that all legs were visible from at least one pair of cameras, at all times. Individual flies were tethered to a 34-gauge needle by their thorax using ultraviolet-cured glue, and were then placed on an air-supported spherical treadmill (6 mm diameter). Tethered flies were illuminated with a custom infrared ring emitting focused light to the plane of the ball. The ball was tracked at 50 Hz through two orthogonally placed custom motion sensors. The cameras, infrared light source and the ball tracker were all triggered by an Arduino at 200 Hz, with camera exposure time set to 200 µs. Videos were recorded with a resolution of 1,440 × 1,072 pixels.

#### Camera calibration, two-dimensional pose tracking and 3D pose reconstruction

We used DeepLabCut (v.2.2.3, DLC^67^) to track 33 points of interest on the fly body: the notum, two wing hinges and five joints per leg (thorax-coxa, coxa-trocanter, Fe–Ti, tibia-tarsus and the tarsal tip). Separate ResNet-101 neural networks were trained for all cameras (500,000 iterations) except for the three front-facing cameras, which were all handled by the same network (five networks in total). We used roughly 830 manually annotated frames per camera for initial training of all networks (46 frames each from 18 flies) with a test–train split of 95–5%. An additional round of training was needed using roughly 600 frames each (40 frames from 16 flies) before the tracking was satisfactory (error in pixels less than 4 pixels for all networks). The cameras were calibrated using the calibration module in Anipose^68^ (v.1.0.1). We used a precision manufactured ChArUco board^68^ as a calibration target. The board was imaged from all cameras simultaneously at 15 Hz and maximum resolution (1,440 × 1,072). When the board-based calibration alone failed to give satisfactory results (as measured by the mean reprojection error in pixels of the final 3D model output of Anipose being greater than 20 pixels for any point), their animal-based calibration module was used to bring the mean reprojection errors below this threshold. Anipose was used to triangulate all points, as well as to calculate the flexion angle for all four joints from each leg.

#### Ball fitting and swing-stance prediction

Step cycles were estimated based on the proximity of the tracked tarsal tip coordinates to the ball surface. As the ball itself was not tracked, we fit a sphere to the 3D reconstructed tarsal tip coordinates. The position of the sphere in space and its radius was optimized iteratively using the squared distance of the tarsal tips to the surface of the sphere. Tarsal tip positions within 0.05% of the radius were considered as stance, others as swing. Of note, swing and stance phases shorter than 10 ms were filtered out.

#### Activation of halt neurons in tethered flies walking on the ball

To precisely quantify the difference between the halting phenotypes on activation of BRK, FG and BB (Figs. 1g and 4a–d and Extended Data Fig. 3), male flies aged between 7 and 10 days expressing CsChrimson in the respective halt neurons were subjected to optogenetic activation on the ball. The flies were starved for 6–9 h before the experiment to increase the likelihood of high-speed spontaneous walking. The compressed air supplied to the ball was passed through an in-line heating element (Southeastern Heaters and Controls, Inc., Heater FLC-2 120 V 250 W coupled with a TPC10063 controller) to bring the local temperature on the ball up to 32 °C. Each fly was left on the ball for a maximum of 20 min, during which a maximum of ten trials (7 s each) could be triggered in closed-loop with the forward velocity of the fly. Each trial consisted of 2 s of light OFF, followed by 3 s of red light stimulation (66 Hz, roughly 0.04 mW mm^−2^) delivered through an LED-coupled optic fibre (625 nm, Thorlabs M625F2).

#### Kinematic analysis

To quantify the differences between halting phenotypes, we considered only the trials during which flies were walking with an average velocity greater than 1 mm s^−1^ before optogenetic stimulation. The whole dataset was segmented into cases in which the light stimulation onset coincided with a continuing swing phase or stance phase. A stopping bout was defined as when the average ball velocity was below 0.8 mm s^−1^ over a minimum period of 250 ms. Swing duration before optogenetic stimulation was calculated as the median swing duration from all swing events in the prestimulation period. Swing duration after optogenetic stimulation was the duration of the continuing swing at light onset.

#### Activation experiments in tethered, decapitated flies walking on the ball

Flies were ice-anaesthetized, decapitated and their neck was sealed using ultraviolet-cured glue. Only flies that recovered well from decapitation (roughly 95% of all decapitated flies) (that is, showing proper self-righting and spontaneous grooming) were tethered to the 34-gauge needle with ultraviolet-cured glue and placed on the ball. In MDN and MDN + BRK activation experiments (Fig. 4e–j), decapitated flies were subjected to ten trials (7 s each) with 3 s red light stimulation (66 Hz, 0.04 mW mm^−2^). In the case of BDN2 activation in decapitated flies, we observed robust walking when we restricted to testing older flies (10–12 days old, for Fig. 4e–j) flies compared to the 7–10-day-old age range that was used for initial experiments (Extended Data Fig. 6e), probably due to lower and more variable expression levels in younger flies. Further, for BDN2 and BDN2 + BRK experiments (Fig. 4e–j), given expression levels of CsChrimson in BDN2 differed between individuals, for each fly we sampled five different intensities (0.01, 0.015, 0.025, 0.041, 0.058 mW mm^−2^) of stimulation and chose the particular intensity at which that individual fly showed some degree of intention for forward walking (if BDN2 expression is weak, BRK dominates the phenotype and legs do not show any movement). All videos acquired were passed through our 3D pose estimation pipeline as elaborated above. The SIZ was defined as the range below the 25th percentile (for forward walking) or above the 75th percentile (for backward walking) of the Fe–Ti flexion angle at which MDN or BDN2 activated flies initiate swings, respectively. The SIZ count is number of times the Fe–Ti joint angle enters the SIZ in a single trial. The dwell time in SIZ is the time spent by the leg in the SIZ each time it enters it. The percentage of swings in SIZ refers to the number of SIZ events in which the leg performs a swing, divided by total number of SIZ events, per trial.

#### Segment-specific grooming in decapitated flies on the ball

Tethered, decapitated flies expressing GtACR1 in different subsets of the BRK neurons were placed on the ball and acclimated to 45 s green light (530 nm, Thorlabs M530F2, continuous, roughly 0.018 mW mm^−2^) before recording. Subsequently, flies were again exposed to 45 s green light for silencing BRK, while front leg or hind-leg-specific grooming was induced. Hind-leg grooming was induced by gently touching the wing with a brush (Fig. 6p–r), whereas foreleg grooming (Fig. 6.m–o) was induced by touching the front leg with a brush covered with yellow dust (Reactive Yellow 86, catalogue no. sc-296260). These videos were manually scored for grooming bouts. We considered the start of a grooming bout when leg lifted off the ball surface, and the end when the leg touched down the ball surface (in case of stable grooming bouts) or when grooming movements ended (in case of destabilized grooming in which the fly tries to regain normal posture). The 3D pose (front and hind legs) of these flies were reconstructed using the pipeline mentioned above, and the Fe–Ti flexion angle standard deviation during each grooming bout was quantified. Mid-legs could not be tracked due to occlusions with the brush. Ball movement was defined as the sum of the *x*, *y* and *z* rotational velocities of the ball.

### Functional connectivity

For all functional connectivity experiments, tissues were imaged under a Bergamo II two-photon (2P) microscope using a ×20 numerical aperture (NA) 1.0 objective lens (XLUMPLFLN, Olympus). For imaging, GCaMP signal was recorded with a 920 nm Ti:Sapphire laser (MaiTai DeepSee, Newport Spectra-Physics). For optogenetic activation, a fibre-coupled 655 nm LED (FC1-LED, Prizmatix) was positioned with a micromanipulator (Misumi XYZFG2) to deliver pulse trains of red light onto the tissues, with an inter-stimulation interval greater than 10 s. The LED was controlled and synchronized with the resonance imaging scanner (8.3 kHz) using ScanImage software (MBF Bioscience), such that red light stimulation was permitted only during the non-imaging fly-back time of the scanner. This ensured that no light artefact appeared in the region of interest. LED power was measured with a power meter (PM100A, Thorlabs) paired with a photodiode sensor (S121C, Thorlabs), at roughly 1 cm distance between LED and sensor. Background subtracted imaging data were analysed using ImageJ and MATLAB as in ref. ^19^. Change in calcium signal was computed using ∆*F*/*F* = (*F* − *F*_0_)/*F*_0_, where *F*_0_ is the mean fluorescence 2 s period before stimulation onset. Statistical comparisons between groups were performed by quantifying the area under the curve (0–2 s poststimulation).

To test whether BRK is excitatory (as predicted by the connectome), we optogenetically activated BRK while recording calcium activity from its main postsynaptic partner that we called BON1 (Brake Output Neuron 1). In this experiment (Extended Data Fig. 5e,f), whole central nervous systems (brain + VNC) of female flies (6–9 days old) of the genotypes BRK-Gal4>UAS-CsChrimson; BON1-LexA>LexAop-GCaMP6s or +>CsChrimson; BON1>GCaMP6s were dissected and imaged in extracellular saline solution bubbled with carbogen^19^. Tissues were transferred on a poly-l-lysine-coated coverslip fixed in an imaging chamber (ALAMS-518SWPW). During the entire imaging session, bubbled extracellular saline with carbogen was delivered over the brains by means of a perfusion system (78018-40, Masterflex). For BRK activation, 2 s pulse trains of red light (50 Hz, roughly 0.08 mW mm^−2^) were delivered onto the tissues. Simultaneously, single-plane imaging of the BON1 soma was performed at a rate of 6 Hz.

To test whether FG and/or BB receive information from gustatory sensory neurons, we activated Gr5a neurons while recording calcium activity from either FG or BB. In these experiments (Fig. 5b and Extended Data Fig. 8a–d), female flies (6–9 days old) of the genotypes (1) Gr5a-LexA>LexAop-ChrimsonR; FG-Gal4>UAS-GCaMP7b, (2) Gr5a-LexA>LexAop-ChrimsonR; BB-Gal4>UAS-GCaMP7b, (3) +>LexAop-ChrimsonR; FG-Gal4>UAS-GCaMP7b and (4) +>LexAop-ChrimsonR; BB-Gal4>UAS-GCaMP7b were tethered, dissected and imaged as in refs. ^41,69^. In brief, flies were ice-anaesthetized and vertically mounted on chamber. The cuticle was removed from the head to expose the SEZ brain region from where FG and BB neurites could be imaged (single plane, 3 Hz). The front legs were ultraviolet-glued to avoid movements during imaging, and Gr5a neurons were photostimulated by delivering pulsed red light onto the proboscis (100 Hz, roughly 0.08 mW mm^−2^). A side camera (FLIR, SpinView software) ensured reproducible positioning of the LED in front of the proboscis (distance of roughly 1 cm). Flies were either fed or starved 24 h before the experiment (placed in vials with tissue soaked with water and all-*trans* retinal). Experiments for fed versus starved state comparison of FG activity on Gr5a stimulation and corresponding controls, (Extended Data Fig. 8a), were performed on a setup described in ref. ^41^, which used a 0.66 Hz imaging frame rate and wide-field opto-stimulation (650 nm) through the imaging objective, instead of the fibre-coupled LED used in all other experiments.

### Muscle imaging

To test the influence of BRK, FG or BB activation on leg muscle activity, we performed one-photon (1P) and 2P calcium imaging of front-leg femoral muscles (Fig. 4 and Extended Data Fig. 7). Female flies (5–8 days) of the genotypes (1) BRK-Gal4>UAS-CsChrimson; MHC-LexA>LexAop-GCaMP6f (ref. ^38^), (2) FG-Gal4>UAS-CsChrimson; MHC-LexA>LexAop-GCaMP6f, (3) BB-Gal4>UAS-CsChrimson; MHC-LexA>LexAop-GCaMP6f and (4) +>UAS-CsChrimson; MHC-LexA>LexAop-GCaMP6f were cold anaesthetized and placed on a circular coverslip fixed in an imaging chamber (CSC-25L, Bioscience Tools). The wings and five legs, except the left front leg, were ablated. We applied ultraviolet-cured glue around the fly body and on the proboscis to avoid movements during imaging. The remaining front leg was glued either in its flexed (Fe–Ti angle roughly 10–27°) or extended (Fe–Ti angle roughly 123–147°) position. The fly holder was then flipped, such that the fly was underneath the coverslip on the opposite side to the objective. We could then add water on the coverslip and image with a water immersion objective (×20 NA 1.0 objective lens, Olympus XLUMPLFLN), while the fly remained dry on the other side of the coverslip. Muscle GCaMP6f signal in the front leg was recorded using 1P, wide-field fluorescence imaging (488 nm mounted LED; Thorlabs) and then the same sample was imaged under 2P imaging (920 nm Ti:Sapphire laser; MaiTai DeepSee, Newport Spectra-Physics). Under 2P conditions, because the fly was in complete darkness, control flies often showed light responses to the opto-stim LED (note that even though we use 655 nm LED, it is likely that there was a dim tail of the LED spectrum that is present in the visual spectrum of the fly). On the other hand, under the 1P condition, because of bright blue imaging light, control flies did not show any responses to the 655 nm opto-stim LED. Hence we used 1P data for analysis, but still continued performing 2P imaging given it was very useful to draw the muscle boundaries and also depict the imaging videos. For activating CsChrimson expressed in BRK, FG or BB, the 655 nm LED described above was placed with a micromanipulator and used to deliver red light towards the fly thorax (roughly 1 cm distance). A given session typically consisted of four stimulations for both 1P and 2P experiments. Change in calcium signal was computed using ∆*F*/*F* = (*F* − *F*_0_)/*F*_0_, where *F*_0_ is the tenth percentile fluorescence intensity level in the 2 s period before stimulation onset. For ∆*F*/*F* calculation, all four stimulation trials were considered and averaged across the different sessions.

In 1P experiments (Fig. 4k and Extended Data Fig. 7d), red light stimulation (continuous, roughly 0.01 mW mm^−2^) was delivered for 1.6 s with 10 s inter-stimulation intervals; muscle GCaMP6f signal was acquired at a frame rate of 50 Hz. Red light stimulation was controlled by means of transistor–transistor logic inputs synchronized to the imaging session using ThorCam software (Thorlabs) plus an external Arduino based trigger box (Thorlabs TSI-IOBOB2). In 2P experiments (Fig. 4l), 2 s of red light stimulation (100 Hz, roughly 0.08 mW mm^−2^) was as described in functional connectivity experiments; muscle GCaMP6f signal was acquired at 6 Hz.

To probe the effect of halting neurons on spontaneous muscle activity, we performed a separate set of experiments (Extended Data Fig. 7a,b,e,f) in which red light stimulation was delivered for roughly 2 s only during high muscle baseline activity.

For tibia-movement experiments (Extended Data Fig. 7c), we used a protocol described in ref. ^70^. Briefly, a fly was mounted on a coverslip as described above. A magnetic pin (Entomoravia, Austerlitz Insect Pins; 1 mm length; 0.1 mm diameter) was then glued on the front left leg tibia. A magnet mounted on a programmable servo motor (Silver max Hybrid Servo Motor, Precise Motion and Control Inc.) was used to forcibly flex and extend the Fe–Ti joint (1 s per flexion or extension, repeated four times). Femoral muscle activity was imaged under epifluorescence (1P) while delivering optogenetic stimulation using the 655 nm LED described above, during the entire session (continuous, roughly 0.08 mW mm^−2^). Muscle GCaMP signal was acquired at a frame rate of 50 Hz. A simultaneous video of the tibia movements (720 × 540 resolution; 50 Hz) was acquired in SpinView software (FLIR) and synchronized to the imaging session using ThorCam software (Thorlabs) plus an external Arduino based trigger box (Thorlabs TSI-IOBOB2). Change in calcium signal was computed using ∆*F*/*F* = (*F* − *F*_0_)/*F*_0_, where *F*_0_ is the median fluorescence 300 ms period before movement initiation onset.

### In vivo imaging

For imaging BRK activity in vivo (Fig. 6), female flies (4–7 days) of the genotypes BRK-Gal4>Act88F:Rpr; UAS-GCaMP6f; UAS-tdTomato or BDN2-Gal4>Act88F:Rpr; UAS-GCaMP6f; UAS-tdTomato were anaesthetized on ice and tethered on a custom fly holder^19^. Thoracic dissection for VNC imaging was then performed as described in ref. ^15^. Artificial Haemolymph (AHL^71^) solution was used during dissection and imaging of the exposed VNC. After dissection, the holder was placed under a Bergamo II 2P microscope (Thorlabs) under a water immersion objective (×40 NA 0.8 objective lens Nikon CFI APO near infrared). An air-supported ball was positioned under the fly in a similar setup as described above for leg kinematics analysis. Temperature under objective (roughly 25–30 °C) was controlled and maintained by using a heater (Southeastern Heaters & Controls, Inc., FLC-2 120 V 250 W paired with a TPC10063 controller) paired to the air–ball system. Flies with uncoordinated leg movements (roughly 25%) were discarded before the experiment. After an acclimation period to the ball (roughly 15–20 min), a volume containing axonal projections from BRK or BDN2 was imaged using at a volumetric rate of 2 Hz (BRK) or 6 Hz (BDN2), using a 920 nm Ti:Sapphire laser and a fast *z*-piezo device. Video of the behaving fly (720 × 540 resolution; 200 Hz) was acquired in SpinView software (FLIR) and synchronized to the imaging session using ScanImage software (MBF Bioscience). The synchronized calcium imaging and ball velocity data were analysed offline. For BRK imaging experiments, additional behaviours were manually annotated (Fig. 6e–h) using FlyTracker software^63^. Data analysis was performed using custom scripts in Python and MATLAB.

### Immunohistochemistry

All central nervous system dissections and immunohistochemistry were performed as described in ref. ^72^ with detailed protocols available at https://www.janelia.org/project-team/flylight/protocols. Primary antibodies used were chicken anti-GFP (1:1,000, Thermo Fisher Scientific, AB_2534023), rabbit anti-dsRed (1:500, CloneTech, AB_10013483) and anti-Bruchpilot (1:500, nc82, mouse monoclonal, Developmental Studies Hybridoma Bank, AB_2314866). Alexa fluor secondary antibodies (Thermo Fisher Scientific) were used at 1:500 dilution (Goat antichicken, Alexa488, AB_2576217; Goat antirabbit, Alex568, AB_10563566; Goat antimouse, Alex568, AB_2534072 and Goat antimouse, Alex647, AB_141725).

### FISH

This was performed as a part of a large-scale fluorescence in situ hybridization (FISH) imaging session by A. Petruncio and the FlyLight team at the Janelia Research Campus, as described in ref. ^73^.

### Connectome-constrained modelling

The neuronal activity was simulated as a spiking neural network in the brian2 software v.2.5.1 (ref. ^74^). Model details, including the original code, are described elsewhere^7^. The original code was modified to allow for stimulating and silencing neurons at arbitrary time points throughout the simulation. Briefly, a leaky integrate-and-fire model was constructed based on the fully annotated connectome^8^. The connection strength between two neurons was set as proportional to the number of synapses linking them. Neurotransmitter predictions based on the EM dataset^35,75^ determine whether the interaction between two neurons is inhibitory (GABA, Glu) or excitatory (all other). Neuronal stimulation was mimicked in the model by adding a Poisson spike train of defined frequency as an input to a neuron. Neuronal silencing was mimicked by simply severing all outgoing synaptic connections of the desired neuron. It is important to realize that the intrinsic firing rate of neurons in this model is zero and that the Poisson inputs constitute the only external input. The firing rates reported here are averaged over 30 trials, for 1,000 ms each with 0.05 ms integration time steps.

The walk neurons P9 and BPN were stimulated bilaterally at 150 and 50 Hz, respectively. The firing rates for the top 100 neurons are shown in Fig. 3a,e. Figure 3b–d,f–h shows the same neurons as Fig. 3a,e, but here the stop neurons BB, FG and BRK, respectively, are stimulated at 150 Hz between 0.25 and 0.75 s. All rates in the top Fig. 3a–h were smoothed with a Gaussian kernel (sigma 25 ms). In the wiring diagram shown in the bottom of Fig. 3a–h, only the most active DNs with firing rates greater than 10.3 and 20 Hz for P9 and BPN stimulations are shown. The node colour shows the average firing rates of the most active DNs during walk neuron stimulation (Fig. 3a,e) and during walk–halt costimulation (Fig. 3b–d,f–h) normalized to the maximum firing rate in Fig. 3a,e, respectively. As BPNs project contralaterally, the left hemisphere DNs receiving input from contralateral BPNs (right) were chosen for representation (this applies to Extended Data Fig. 10a,b, in which walk neurons BPNs and P9 were stimulated at 150 and 50 Hz, respectively, and oviDN and MAN1 were stimulated at 150 Hz).

Extended Data Fig. 6b shows all neurons that were differentially affected in walk neuron stimulation and walk neuron–stop neuron costimulation. Walk neurons BPN and P9 were stimulated at 150 and 50 Hz, respectively. Stop neurons FG and BB were stimulated at 150 Hz.

The left hemisphere GRNs were activated (150 Hz) in Fig. 5a and the downstream feeding pathway with increased firing rates, along with FG and BB, are shown as a wiring diagram. The node colours correspond to the firing rate, except for oDN1 and BDN1 (for simplicity, the graphs are limited to one hemisphere). The heatmaps in Fig. 5c,d show the average firing rate of oDN1 and BDN2 normalized to the maximum value in the left panel, respectively. The left shows costimulation of left hemisphere sugar GRNs and bilateral P9 or BPN walk neurons. The middle shows the same as left, but while silencing stop neuron FG bilaterally. The right shows the difference in firing rate between left and middle panels (this applies to Extended Data Fig. 8e in which only the difference in firing rate for oDN1 and BDN1 in case of BB is shown). The heatmap in Extended Data Fig. 9c demonstrates activation of sugar GRNs (left hemisphere) and eye bristles at 150 Hz and the resultant change in firing of FG, BB and DNg12 neurons (grooming command neurons). Colours and values on the heatmap indicate their firing rates.

Cytoscape^75^ (v.3.10.0) was used to create all the wiring diagrams shown in this study. The edges in Figs. 3a–h and 5a and Extended Data Figs. 2b, 9b,f and 10a–c illustrate the connectivity in the model (red denotes excitatory, blue denotes inhibitory). The arrow size represents the connection strength (connections below five synapses are excluded). For simplicity, only one hemisphere is shown, except in Extended Data Figs. 2b and 9b. The node colours represent firing rates wherever mentioned.

#### Experimental procedures and statistics

We used standard sample sizes, from the field and similar to previous work. All behavioural experiments were reproduced independently at least twice. Control and experimental flies were scored in random order during behavioural experiments. Behavioural experiments were performed with the experimenter blinded to genotype. All statistical tests were performed in MATLAB or Graphpad Prism. All two-group comparisons (unless indicated otherwise) were performed using a non-parametric Mann–Whitney test. All multiple-group comparisons (unless indicated otherwise), were performed using a non-parametric Kruskal–Wallis test followed by Dunn’s multiple comparisons with appropriate controls. Exact sample size values for each plot are reported in Supplementary Table 1.

### Reporting summary

Further information on research design is available in the Nature Portfolio Reporting Summary linked to this article.

## Online content

Any methods, additional references, Nature Portfolio reporting summaries, source data, extended data, supplementary information, acknowledgements, peer review information; details of author contributions and competing interests; and statements of data and code availability are available at 10.1038/s41586-024-07854-7.

## Supplementary information

Reporting Summary

Supplementary Table 1 Experimental genotypes and sample sizes. Excel file including all abbreviated genotypes used in this study, their full experimental genotypes and exact sample sizes, grouped by figure numbers and panels.

Supplementary Table 2 Source of all transgenic flies: Excel file including all strains used in this study, as well as their full genotypes, abbreviations and sources or identifiers.

Supplementary Table 3 Connectome identities. Excel file including all FlyWire/FANC/MANC identifiers for left- and right-hemisphere neurons considered in this study, as well as their community labels, cell types and names given in this study.

Supplementary Video 1 Activation of halt neurons. Transient activation of different halt neurons (BB, FG, BRK) using CsChrimson in untethered (0–7 s, 2× speed) and tethered flies with annotated leg joints (top) and corresponding 3D reconstruction of leg kinematics (bottom) (8–15 s, 0.5× speed), related to Fig. 1 and Extended Data Fig. 3. Red circle on top left indicates light ON (625 nm LED).

Supplementary Video 2 MDN coactivation with halt neurons. Transient activation of MDN (0–6 s) alone or coactivated with BB (7–13 s), FG (14–21 s) and BRK (22–29 s) using CsChrimson in free-walking flies, related to Fig. 2b,e,h,k and Extended Data Fig. 4c Trajectory in blue and pink indicated forward and backward walking resp. Red dot on top left indicates light ON (630 nm LED). Video is being played at 2× speed.

Supplementary Video 3 P9 coactivation with halt neurons. Transient activation of P9 alone or coactivated with different halt neurons (FG, BB, FG+BB) using CsChrimson in free-walking flies, related to Fig. 2. Red dot on top left indicates light ON (630 nm LED).

Supplementary Video 4 BDN2 activity correlates with forward walking. Calcium activity recording (2P) of BDN2 axons in the VNC of a BDN2-Gal4>Act88F:Rpr; UAS-GCaMP6f;tdTomato fly during walking, related to Fig. 3l. Here only showing GCaMP channel with white arrowheads indicating BRK axons (middle), red moving bar on plot (right) shows translational velocity synchronized with fly video (left) and imaging recording.

Supplementary Video 5 BRK activation increases resistance to leg movements. Transient activation of MDN or BDN2 alone (left), and MDN or BDN2 coactivated with BRK (right) using CsChrimson in decapitated, tethered flies with annotated leg joints and corresponding 3D reconstruction of leg kinematics, related to Fig. 4e–j. Red circle on top left indicates light ON (625 nm LED). Video is being played at 0.5× speed.

Supplementary Video 6 BRK activation drives a resistance-like state in the femoral muscles. Calcium activity recording (intact: 1P from 0 to 15 s, 2P from 16 to 30 s) of femoral muscles in the front leg of MHC-LexA>LexAOP-GCaMP6f; BRK-Gal4>UAS-CsChrimson flies with their Fe–Ti joint forcibly kept (glued) in extended (left) or flexed position (right), related to Fig. 4k–n. Red dot indicates light ON (655 nm LED), filled arrowheads show muscle activity on BRK activation, empty arrowheads indicate inactive muscles. Video is being played at 0.5× speed.

Supplementary Video 7 BRK activation leads to inhibition of highly active muscles and activation of specific muscles to reach resistance state-like activity profile. Calcium activity recording (2P) of femoral muscles in the front leg of MHC-LexA>LexAOP-GCaMP6f; BRK-Gal4>UAS-CsChrimson flies with their Fe–Ti joint forcibly kept (glued) in extended (left) or flexed position (right), related to Extended Data Fig. 7a,b. Red dot indicates light ON (655 nm LED), filled arrowheads show muscle activity on BRK activation, empty arrowheads show inactive muscles/inhibition of prestimulation muscle activity. Video is being played at 0.5× speed.

Supplementary Video 8 FG and BB are recruited for halting in the context of feeding. Silencing of halt neurons (BB, FG and BRK) using GtACR1 in food-blob interaction assay showing distinct phenotype on food/sucrose encounter, related to Fig. 5f–h. Green dot at top left indicates light ON (540 nm LED).

Supplementary Video 9 BRK is recruited for halting during grooming. Calcium activity recording (2P) of BRK axons in the VNC of BRK-Gal4>Act88F:Rpr; UAS-GC6f tdTomato flies during front-leg rubbing (Fly1, left) and hind-leg rubbing (Fly2, right), related to Fig. 6a–h. Coloured squares indicate onset of rubbing events, white arrowheads indicate segment-specific BRK activity. Video is being played at 0.2× speed.

Supplementary Video 10 Silencing BRK destabilizes grooming flies. Transient inhibition of BRK (GtACR1) in grooming intact flies (from 0 to 7 s) and decapitated flies (from 8 to 22 s), compared to control flies, related to Fig. 6i–l. Green dot at top left indicates light ON (540 nm LED).

Supplementary Video 11 BRK is recruited in a segment-specific manner for stabilization during grooming. Induction of hind leg/wing grooming (top) or front-leg grooming (bottom) in tethered decapitated flies in which different subsets of BRK neurons are silenced using GtACR1. Segment-specific grooming is induced by powdering the specific segment using a brush. Green light for optogenetic silencing is delivered continuously for 45 s before recording, and is continued throughout the trial lasting 45 s, related to Fig. 6m–r. Video is being played at 0.4× speed.

## Source data

Source Data Fig. 1

Source Data Fig. 2

Source Data Fig. 3

Source Data Fig. 4

Source Data Fig. 5

Source Data Fig. 6

Source Data Extended Data Fig. 1

Source Data Extended Data Fig. 2

Source Data Extended Data Fig. 3

Source Data Extended Data Fig. 4

Source Data Extended Data Fig. 5

Source Data Extended Data Fig. 6

Source Data Extended Data Fig. 7

Source Data Extended Data Fig. 8

Source Data Extended Data Fig. 9

Source Data Extended Data Fig. 10

## Data Availability

Data corresponding to all figures are available at the following open source repository: 10.17617/3.OIX8RZ. Source data are provided with this paper.

## Code availability

Custom scripts were written in either MATLAB 2021a or Python. All code related to this paper is available at GitHub (https://github.com/bidaye-lab/Sapkal_et_al_2024.git).
