# Supplementary material for: Neural circuit mechanisms underlying context-specific halting in Drosophila
Source: Nature. 2024 Oct 2;634(8032):191–200. doi: 10.1038/s41586-024-07854-7 (PMC11446846; doi:10.1038/s41586-024-07854-7)
Supplement: Supplementary file 1 — Reporting Summary [file 41586_2024_7854_MOESM1_ESM.pdf]

nature portfolio

Corresponding author(s): Salil S Bidaye

Last updated by author(s): Jun 12, 2024

Reporting Summary

Nature Portfolio wishes to improve the reproducibility of the work that we publish. This form provides structure for consistency and transparency in reporting. For further information on Nature Portfolio policies, see our Editorial Policies and the Editorial Policy Checklist.

Statistics

For all statistical analyses, confirm that the following items are present in the figure legend, table legend, main text, or Methods section.

n/a	Confirmed	
☐	☒	The exact sample size (*n*) for each experimental group/condition, given as a discrete number and unit of measurement
☐	☒	A statement on whether measurements were taken from distinct samples or whether the same sample was measured repeatedly
☐	☒	The statistical test(s) used AND whether they are one- or two-sided<br>*Only common tests should be described solely by name; describe more complex techniques in the Methods section.*
☐	☒	A description of all covariates tested
☐	☒	A description of any assumptions or corrections, such as tests of normality and adjustment for multiple comparisons
☐	☒	A full description of the statistical parameters including central tendency (e.g. means) or other basic estimates (e.g. regression coefficient) AND variation (e.g. standard deviation) or associated estimates of uncertainty (e.g. confidence intervals)
☐	☒	For null hypothesis testing, the test statistic (e.g. *F*, *t*, *r*) with confidence intervals, effect sizes, degrees of freedom and *P* value noted<br>*Give P values as exact values whenever suitable.*
☒	☐	For Bayesian analysis, information on the choice of priors and Markov chain Monte Carlo settings
☒	☐	For hierarchical and complex designs, identification of the appropriate level for tests and full reporting of outcomes
☒	☐	Estimates of effect sizes (e.g. Cohen's *d*, Pearson's *r*), indicating how they were calculated

Our web collection on statistics for biologists contains articles on many of the points above.

Software and code

Policy information about availability of computer code

Data collection	FLIR cameras with SpinView data acquisition software (version 2.4.0.144) were used for recording all behavioral videos. Cameras, LEDs and other electronic components were synchronized using custom built Ardruino based triggering using custom scripts in Ardruino IDE (version 1.8.15). Ball-tracking data was acquired using custom Matlab scripts (R2021a). All two-photon imaging and synchronization was performed using Scanimage software (version 2021, MBF) and the VIDRIO Data Acquistion Board (MBF). All one-photon wide field fluorescence imaging and synchronization was performed using ThorCam software. All immunohistochemistry images were acquired on a Zeiss LSM 880 confocal microscope using ZEN software.<br>Connectomics: Identification of neurons of interest in the connectome data used the Navis Python library, Natverse R library (1.8.22), the online Flywire Gateway tool (https://flywiregateway.pniapps.org/) and the online NeuronBridge tool (https://neuronbridge.janelia.org/). The whole brain connectome based leaky integrate and fire model is described in Shiu et al and publicly available at https://github.com/philshiu/Drosophila_brain_model/tree/main For this work we modified the code to allow silencing and stimulating neurons for arbitrary time periods. This modified version is available at https://github.com/bidaye-lab/Sapkal_et_al_2024.git

Data analysis

Free-walking videos were tracked using FlyTracker (version 1.03) in a Matlab Environment. Tethered walking videos were first tracked using DeepLabCut (version 2.2.3), for 2D tracking per camera. Then 3D pose was calculated using Anipose package (version 1.0.1). The free walking data and 3D tracking data was then processed and analyzed using custom Matlab and Python scripts. One-photon and Two-photon functional imaging data was processed using custom scripts in Matlab, Python and ImageJ. Statistical analysis was performed in Graphpad Prism (version 10.1.2).
Connectomics modeling data was plotted using custom Python scripts. Connectivity diagrams were plotted using Cytoscape software (version 3.10.1).
All analysis scripts are avilable at https://github.com/bidaye-lab/Sapkal_et_al_2024.git

For manuscripts utilizing custom algorithms or software that are central to the research but not yet described in published literature, software must be made available to editors and reviewers. We strongly encourage code deposition in a community repository (e.g. GitHub). See the Nature Portfolio guidelines for submitting code & software for further information.

Data

Policy information about availability of data

All manuscripts must include a data availability statement. This statement should provide the following information, where applicable:

- Accession codes, unique identifiers, or web links for publicly available datasets
- A description of any restrictions on data availability
- For clinical datasets or third party data, please ensure that the statement adheres to our policy

All data related to this manuscript is available at https://doi.org/10.17617/3.OIX8RZ
The data can be analyzed and plotted using scripts provided at https://github.com/bidaye-lab/Sapkal_et_al_2024.git

Research involving human participants, their data, or biological material

Policy information about studies with human participants or human data. See also policy information about sex, gender (identity/presentation), and sexual orientation and race, ethnicity and racism.

Reporting on sex and gender

Use the terms sex (biological attribute) and gender (shaped by social and cultural circumstances) carefully in order to avoid confusing both terms. Indicate if findings apply to only one sex or gender; describe whether sex and gender were considered in study design; whether sex and/or gender was determined based on self-reporting or assigned and methods used.
Provide in the source data disaggregated sex and gender data, where this information has been collected, and if consent has been obtained for sharing of individual-level data; provide overall numbers in this Reporting Summary. Please state if this information has not been collected.
Report sex- and gender-based analyses where performed, justify reasons for lack of sex- and gender-based analysis.

Reporting on race, ethnicity, or other socially relevant groupings

Please specify the socially constructed or socially relevant categorization variable(s) used in your manuscript and explain why they were used. Please note that such variables should not be used as proxies for other socially constructed/relevant variables (for example, race or ethnicity should not be used as a proxy for socioeconomic status).
Provide clear definitions of the relevant terms used, how they were provided (by the participants/respondents, the researchers, or third parties), and the method(s) used to classify people into the different categories (e.g. self-report, census or administrative data, social media data, etc.)
Please provide details about how you controlled for confounding variables in your analyses.

Population characteristics

Describe the covariate-relevant population characteristics of the human research participants (e.g. age, genotypic information, past and current diagnosis and treatment categories). If you filled out the behavioural & social sciences study design questions and have nothing to add here, write "See above."

Recruitment

Describe how participants were recruited. Outline any potential self-selection bias or other biases that may be present and how these are likely to impact results.

Ethics oversight

Identify the organization(s) that approved the study protocol.

Note that full information on the approval of the study protocol must also be provided in the manuscript.

Field-specific reporting

Please select the one below that is the best fit for your research. If you are not sure, read the appropriate sections before making your selection.

☒ Life sciences ☐ Behavioural & social sciences ☐ Ecological, evolutionary & environmental sciences

For a reference copy of the document with all sections, see nature.com/documents/nr-reporting-summary-flat.pdf

Life sciences study design

All studies must disclose on these points even when the disclosure is negative.

Sample size

We used standard values from the field and similar to our previous papers (Bidaye et al, Science 2014, Bidaye et al, Neuron 2020, Shiu et al, accepted at Nature, 2024)

Data exclusions	No data was excluded after processing using criteria described in Methods.
Replication	All behavioral experiments were reproduced independently at least twice.
Randomization	Control and experimental flies were scored in random order during behavioral experiments.
Blinding	Behavioral experiments were performed with the experimenter blinded to genotype.

Reporting for specific materials, systems and methods

We require information from authors about some types of materials, experimental systems and methods used in many studies. Here, indicate whether each material, system or method listed is relevant to your study. If you are not sure if a list item applies to your research, read the appropriate section before selecting a response.

Materials & experimental systems

n/a	Involved in the study
☐	☒ Antibodies
☒	☐ Eukaryotic cell lines
☒	☐ Palaeontology and archaeology
☐	☒ Animals and other organisms
☒	☐ Clinical data
☒	☐ Dual use research of concern
☒	☐ Plants

Methods

n/a	Involved in the study
☒	☐ ChIP-seq
☒	☐ Flow cytometry
☒	☐ MRI-based neuroimaging

Antibodies

Antibodies used	chicken anti-GFP (1:1000, Thermo Fisher Scientific, AB_2534023), rabbit anti-dsRed (1:500, CloneTech, AB_10013483) and anti-Bruchpilot (1:500, nc82, mouse monoclonal, Developmental Studies Hybridoma Bank, AB_2314866). Alexa fluor secondary antibodies (Thermo Fisher Scientific) were used at 1:500 dilution (Goat anti-chicken: Alexa488, AB_2576217, Goat anti-rabbit: Alex568, AB_10563566, Goat anti-mouse: Alex568, AB_2534072 and Goat anti-mouse: Alex647, AB_141725).
Validation	Only standard validated antibodies were used as per Janelia Research Campus protocol https://www.janelia.org/project-team/flylight/protocols

Animals and other research organisms

Policy information about studies involving animals; ARRIVE guidelines recommended for reporting animal research, and Sex and Gender in Research

Laboratory animals	Drosophila melanogaster flies (invertebrate species) were used for all experiments. Detailed genotypes, age, sex and strain are described in Supplementary Tables 1 and 2.
Wild animals	No wild animals were used in this study
Reporting on sex	No sexual dimorphism was observed in the halting phenotypes that is the focus of this study. Female flies were used for free walking experiments, functional imaging experiments and immunohistochemistry consistent with previous studies and female fly connectomics data. Male flies were chosen for hgih-resolution leg kinematics experiments because their smaller size was better suited to the high resolution camera field-of-view necessary for leg joint tracking.
Field-collected samples	No field-collected samples were used in this study
Ethics oversight	No ethics approval or oversight is necessary for Drosophila experiments.

Note that full information on the approval of the study protocol must also be provided in the manuscript.

Plants

Seed stocks	*Report on the source of all seed stocks or other plant material used. If applicable, state the seed stock centre and catalogue number. If plant specimens were collected from the field, describe the collection location, date and sampling procedures.*
Novel plant genotypes	*Describe the methods by which all novel plant genotypes were produced. This includes those generated by transgenic approaches, gene editing, chemical/radiation-based mutagenesis and hybridization. For transgenic lines, describe the transformation method, the number of independent lines analyzed and the generation upon which experiments were performed. For gene-edited lines, describe the editor used, the endogenous sequence targeted for editing, the targeting guide RNA sequence (if applicable) and how the editor was applied.*
Authentication	*Describe any authentication procedures for each seed stock used or novel genotype generated. Describe any experiments used to assess the effect of a mutation and, where applicable, how potential secondary effects (e.g. second site T-DNA insertions, mosiacism, off-target gene editing) were examined.*
